# Supplementary material for: Complete Connectomic Reconstruction of Olfactory Projection Neurons in the Fly Brain
Source: Curr Biol. 2020 Aug 17;30(16):3183–3199.e6. doi: 10.1016/j.cub.2020.06.042 (PMC7443706; doi:10.1016/j.cub.2020.06.042)
Supplement: Document S1. Figures S1–S7 and Supplemental References [file mmc1.pdf]

Current Biology, Volume *30*

Supplemental Information

Complete Connectomic Reconstruction of Olfactory Projection Neurons in the Fly Brain

Alexander S. Bates, Philipp Schlegel, Ruairi J.V. Roberts, Nikolas Drummond, Imaan F.M. Tamimi, Robert Turnbull, Xincheng Zhao, Elizabeth C. Marin, Patricia D. Popovici, Serene Dhawan, Arian Jamasb, Alexandre Javier, Laia Serratosa Capdevila, Feng Li, Gerald M. Rubin, Scott Waddell, Davi D. Bock, Marta Costa, and Gregory S.X.E. Jefferis

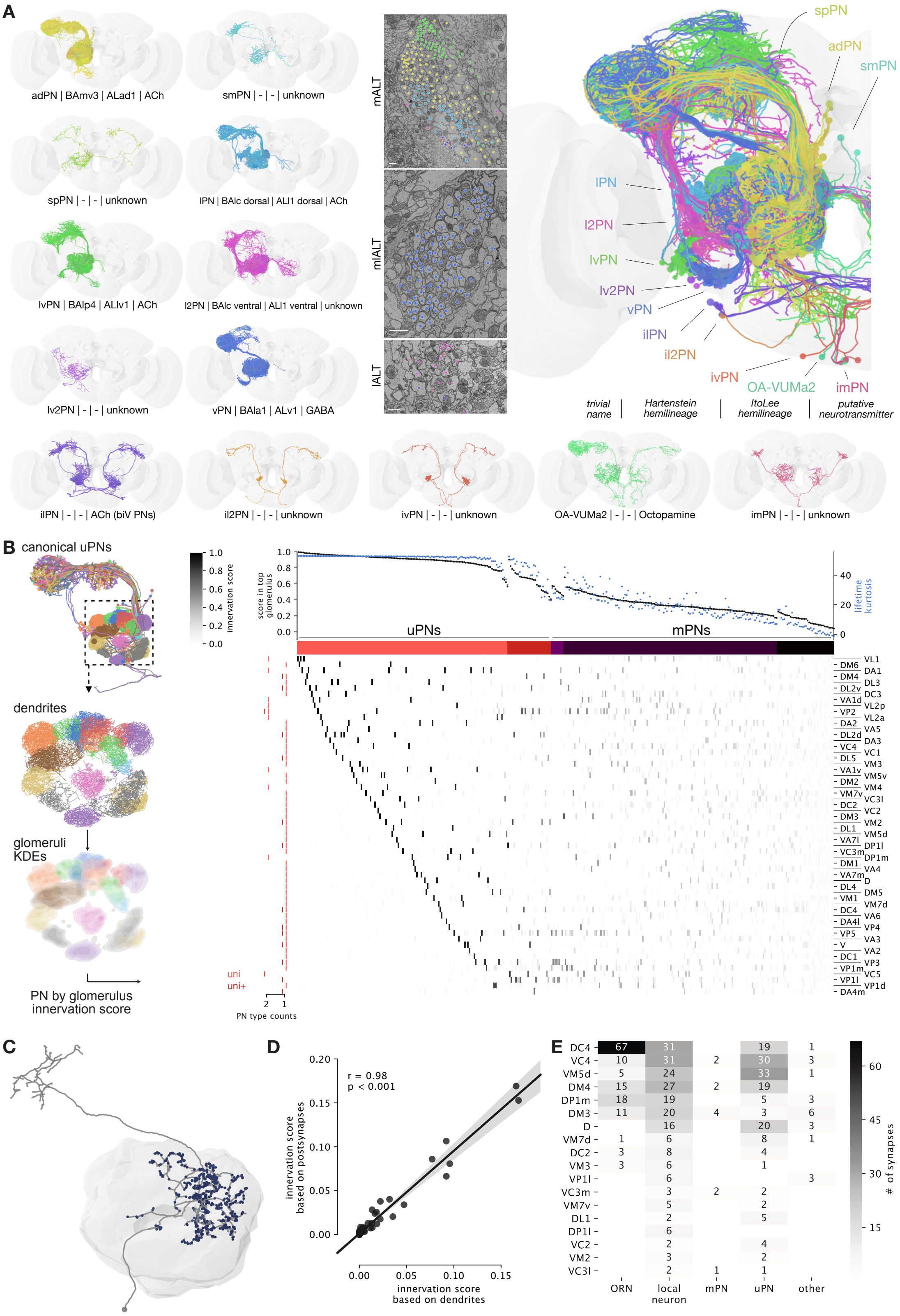

Figure S1: PN lineages, putative transmitters and AL innervation patterns, **Related to to Figure 1.** **A** Projection neurons (PNs) by trivial name; if available, (hemi-) lineage and putative neurotransmitters are given [S1–S4]. EM images show cross sections through the three main antennal lobe (AL) tracts (ALTs) with PN profiles highlighted (scale bar 1 micron). See also Video S1. Abbreviations: d=dorsal; i=inferior; l=lateral; m=medial; p=posterior; v=ventral. **B** PN by glomerulus innervation matrix based on probabilistic model of the AL (see Methods for details). PNs (columns) are sorted and colored according to their clustering in Figure 1E. Top scatter plot shows two metrics for PN sparseness: top innervation score (the highest value in each column) & lifetime kurtosis [S5]. Left line plot shows number of uPN (uni/uni+) types per glomerulus. **C** Single multiglomerular PN (mPN) which was traced to synapse completion in the AL. Dendritic synaptic inputs highlighted in blue. **D** Correlation of innervation score (which is based on only neural cable) and the actual synaptic inputs of the mPN in C. The strong correlation validates use of neurites as proxy for glomerular innervation (Pearson R, $p < 0.001$). **E** Synaptic inputs to dendrites of mPN in C by glomerulus and neuron type. Composition of inputs varies from glomerulus to glomerulus.

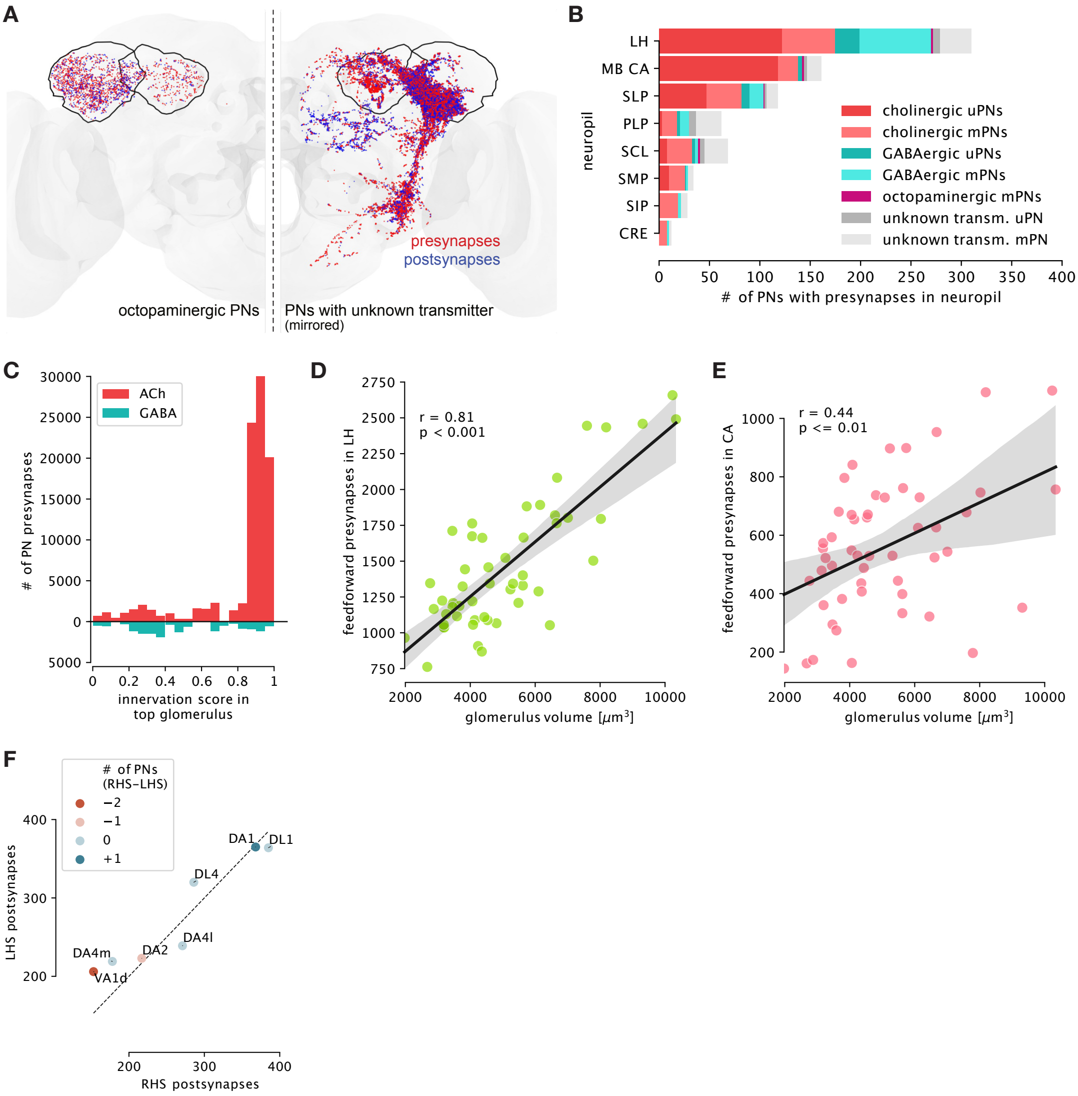

Figure S2: Third-order targets of olfactory feedforward drive, **Related to Figure 2. A** Spatial distribution of presynapses (out-puts, red) and postsynapses (inputs, blue) by octopaminergic PNs and PNs with unknown neurotransmitter. **B** Total number of PNs with >10 presynapses in target neuropil (top 8 shown). The LH is the main target of non-cholinergic and multi-glomerular PNs. **C** Choliner-gic versus GABAergic feedforward axonic presynapses by sparseness. Sparse PNs make up the majority of cholinergic presynapses while GABAergic presynapses stem from a mix of sparse and broad PNs. **D, E** Glomerulus volume versus feedforward presynapses in LH (D) and CA (E). Correlation is stronger for the LH than the CA (Pearson correlation, see STAR Methods for details). **F** Number of postsynapses between left- (LHS) and right-hand-side (RHS) homologs of 7 uPN types.

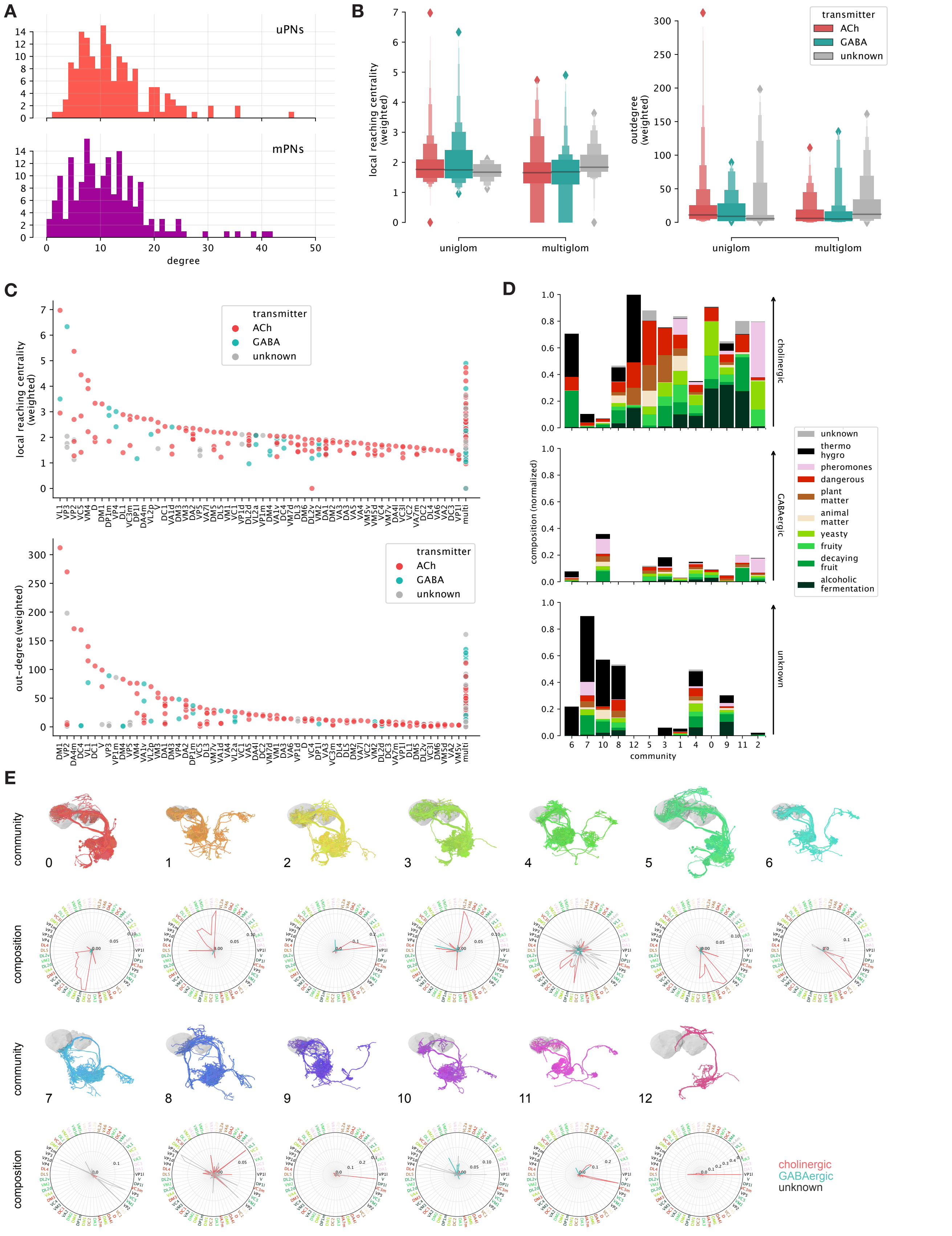

Figure S3: Axo-axonic PN network, Related to Figure 3. A Degree (number of all incoming and outgoing edges) distribution for uni- (top) and multiglomerular (bottom) projection neurons (PNs). **B** Local reaching centrality (LRC, top) and outdegree (bottom) does not differ between PN classes and transmitters. Individual neurons can be strong outliers though (see Figure 3F). **C** LRC (top) and out-degree (bottom) per glomerulus. Each data point represents a single PN (see also Data S3). **D** Normalised composition of axo-axonic PN-PN communities split by neurotransmitter. **E** Morphology and composition by glomerulus of individual communities. Polar plots show density per community per neurotransmitter (i.e. the area under the curves sums up to 1). For example, community 0 consists of mainly cholinergic PNs that most strongly innervate glomeruli DP1m and DM1. Colors of glomeruli labels correspond to odour scene in D.

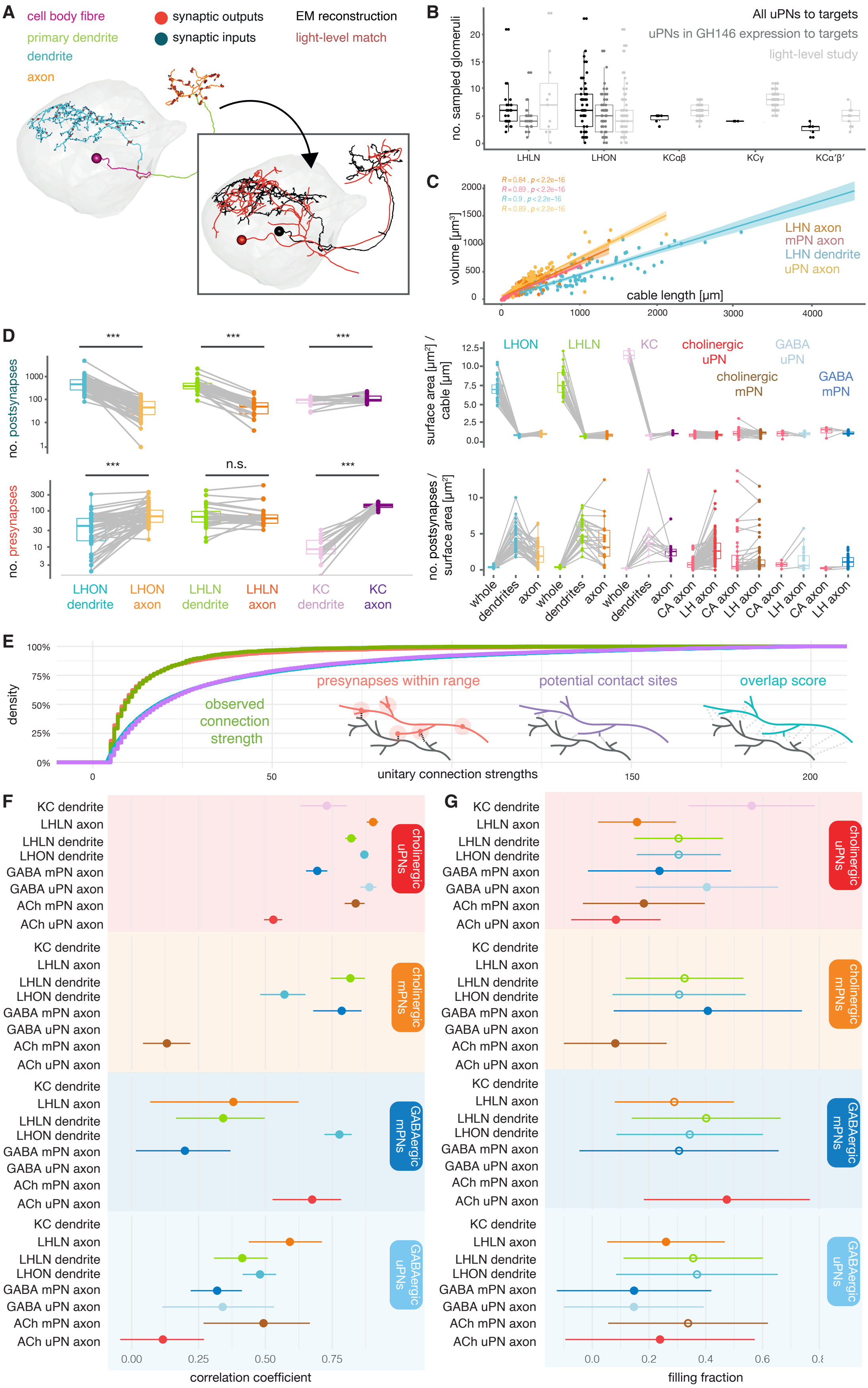

A
cell body fibre
primary dendrite
dendrite
axon
synaptic outputs
synaptic inputs
EM reconstruction
light-level match
B
All uPNs to targets
uPNs in GH146 expression to targets
light-level study
no. sampled glomeruli
LHLN
LHON
KCαβ
KCγ
KCα'β'
C
volume [μm³]
cable length [μm]
LHN axon
mPN axon
LHN dendrite
uPN axon
D
no. postsynapses
no. presynapses
***
n.s.
LHON dendrite
LHON axon
LHLN dendrite
LHLN axon
KC dendrite
KC axon
surface area [μm²] / cable [μm]
LHON
LHLN
KC
cholinergic uPN
GABA uPN
cholinergic mPN
GABA mPN
no. postsynapses / surface area [μm²]
whole
dendrites
axon
CA axon
LH axon
E
density
observed connection strength
presynapses within range
potential contact sites
overlap score
unitary connection strengths
F
G
KC dendrite
LHLN axon
LHLN dendrite
LHON dendrite
GABA mPN axon
GABA uPN axon
ACh mPN axon
ACh uPN axon
cholinergic uPNs
cholinergic mPNs
GABAergic mPNs
GABAergic uPNs
correlation coefficient
filling fraction

Figure S4: Basic anatomical properties of lateral horn neurons, **Related to Figure 4.** **A** Left, an example EM complete recon-struction of an LHON, including its synapses and separated neuron compartments [S6]. Right, this EM skeleton (black) is matched using NBLAST to a reconstructed skeleton from a library of light-level morphologies (red). This library contains defined cell types [S7], and so the EM reconstruction can be identified as AD1a1. **B** Boxplots showing the number of connected glomeruli as assessed from EM reconstruc-tions (black) and light-level studies (light grey). For LHNs, this connectivity comes from a functional assessment of uPN→LHN connections [S8], for KCs light microscopy was used [S9]. Because Jeanne *et al.* [S8] only examined PNs labeled by the driver line GH146, we also restrict our connectivity results to these neurons (dark grey). **C** Correlation between neuronal volume and cable length for dendrites and axons. **D** Right, paired boxplots comparing the numbers of pre- and postsynapses (i.e. output and input synapses) between dendrites and axons for third-order olfactory neurons' dendrites versus their axons (note log10 scale; paired Student's T-tests; *** $p <= 0.001$). Right, the surface areas of different neuronal compartments, and the number of postsynapses per unit surface area. **E** An empirical cumulative density plot displaying the unitary (i.e. single neuron to single neuron) predicted and observed connection strengths for reconstructions in our set (82 LHNs). Inset, schematic for three different ways by which synaptic connectivity may be predicted. 'Overlap score' and 'potential contact sites' do not use any synapse information, 'presynapses within range' uses only the position of output synapses. **F** The Pearson correlation coefficients for the relationship between predicted (from the 'presynapses within synaptic range' approach) and observed connections be-tween neurons. Closed circles, significant correlations ($p <= 0.05$, Student's T-test), open circles, non-significant correlations. **G** The filling fraction, the proportion of predicted synapses that are realised, is shown. Error bars, standard errors of the mean. Closed circles, significant correlations relative to LHON dendrites ($p > 0.05$, Student's T-test), open circles, non-significant correlations. Horizontal, coloured facets indicate the upstream neuron class and compartment, colours indicate downstream neuron class and compartment.

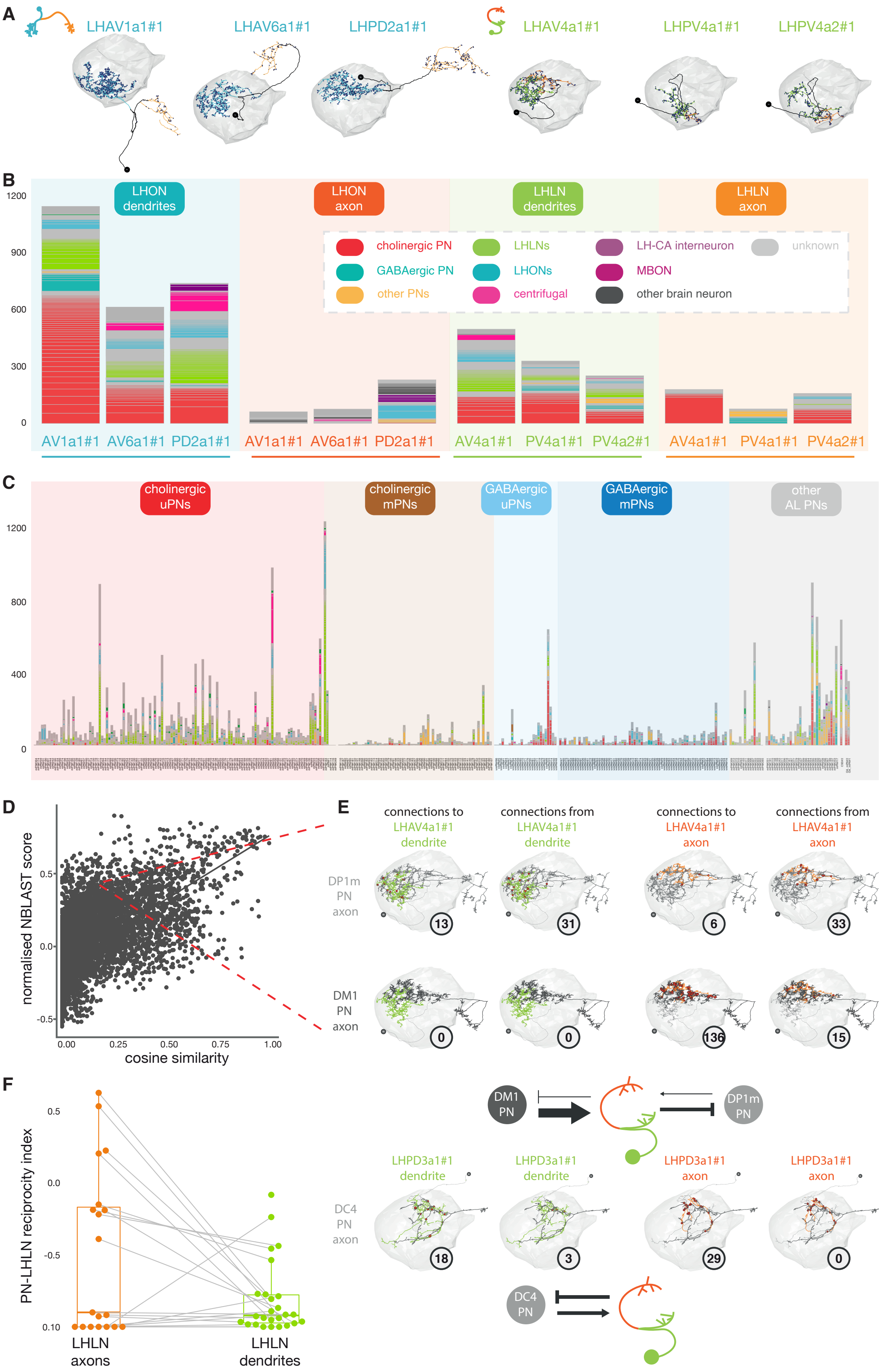

Figure S5: The upstream connectivity of individual neurons in the lateral horn, Related to Figure 5. A Left, the three LHONs chosen for full upstream sampling in the LH. Right, chosen LHLNs. **B** Stacked bar plots showing the result of upstream sampling in the LH for these six neurons, faceted by neuron class and compartment. **C** Stacked bar plots showing the result of upstream sampling all PNs in the LH, faceted by neuron class. **D** Scatter plot showing the correlation between PN-PN morphological similarity (NBLAST) and upstream connectivity similarity. **E** Examples of inhibitory connectivity motifs between neurons fully reconstructed in this study, with connection locations shown in red (occasionally obscure one another). Upper, an example of lateral PN-PN inhibition via an LHLN. Lower, an example of feedback inhibition. Number in circles are total connections. **F** Paired boxplot comparing the degree to which LHLN axons and dendrites reciprocate connections with PN axons. Plots show an index for connection reciprocity between LHLNs and PNs (LHLN→PN-PN→LHLN connectivity/total).

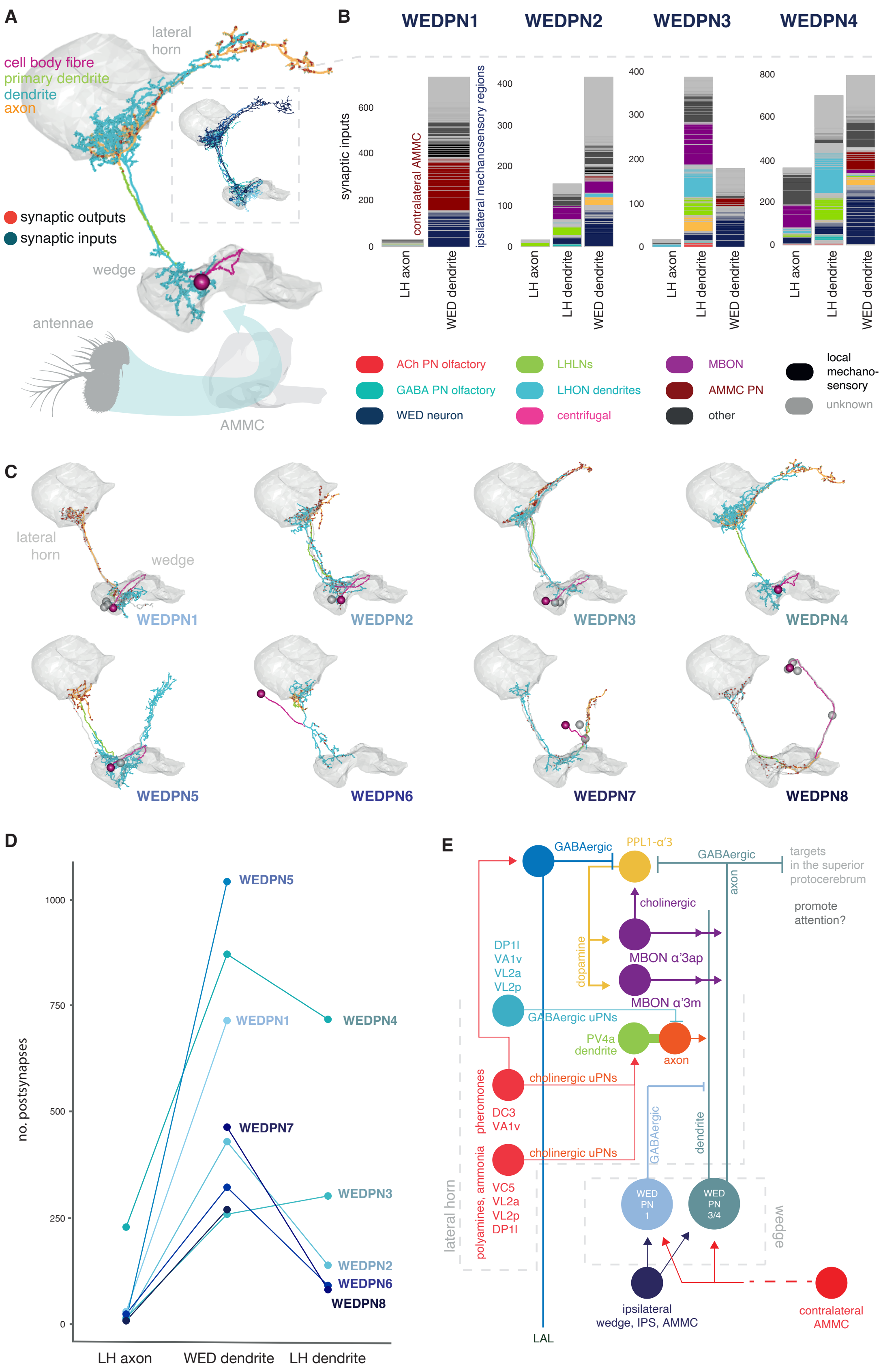

Figure S6: Feedforward wind-sensitive and feedback memory input to the lateral horn, Related to Figure 6. A Exemplary synaptic reconstruction of a WED-PN (a WED-PN4). **B** Stacked bar plots showing the result of upstream tracing from WED-PN compart-ments, for one exemplary neuron for each of four types. **C** WED-PN reconstructions built from the FAFB dataset, broken down into eight cell types. Exemplary fully reconstructed WED-PN shown in colours. **D** The number of synaptic inputs different exemplary WED-PNs, bro-ken down by neuropil and neuron compartment. Cell type colours correspond to the colours of labels in C. **E** Putative circuit integrating the results of lifetime learning, via an MBON, with specific, hardwired olfactory and mechanosensory circuitry.

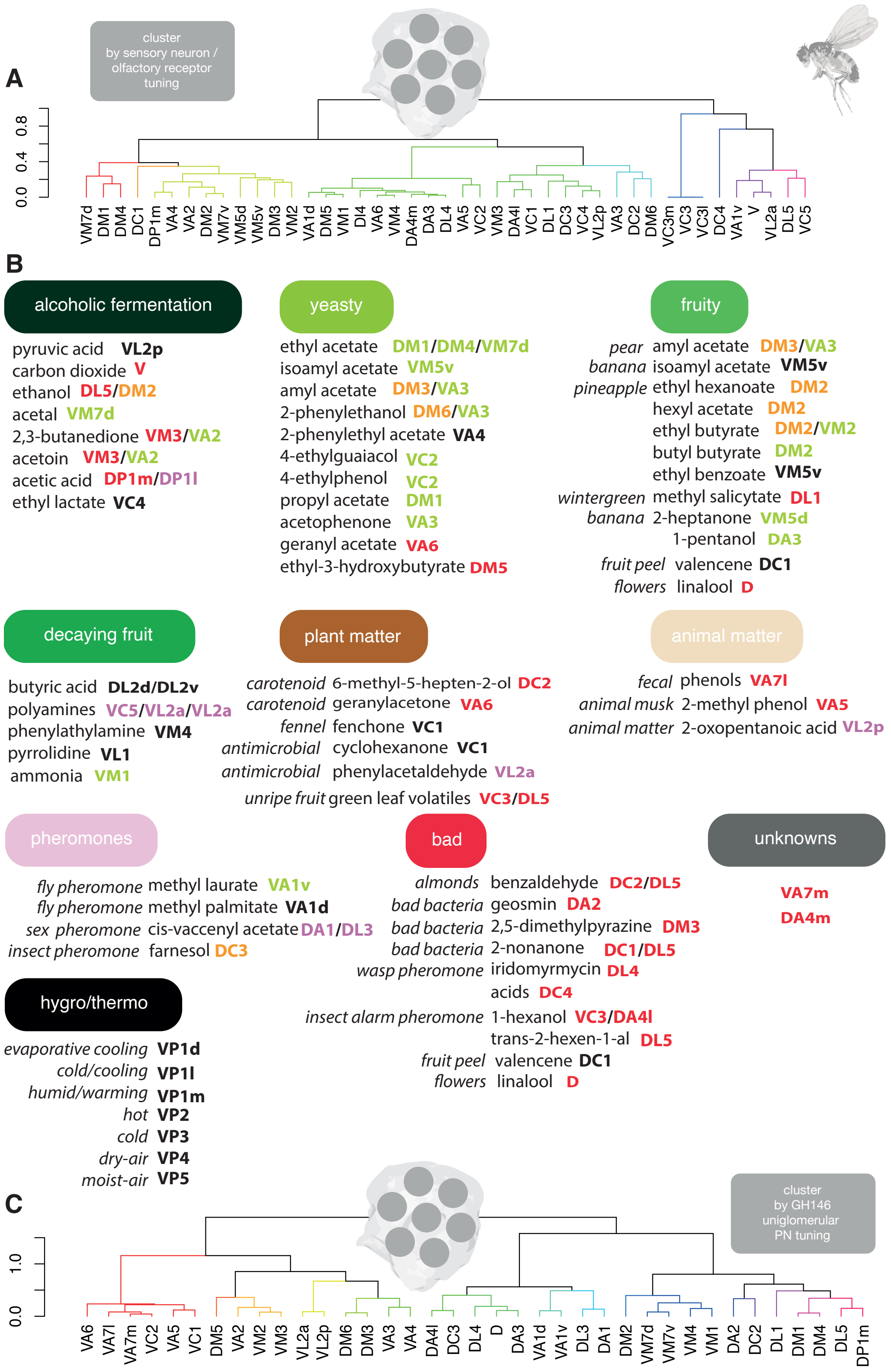

Figure S7: Glomeruli valences and odours scenes, Related to Figures 2 and 6. A Hierarchical clustering (Ward's method, arbitrary height cut off for colouring at 0.3) of manipulated odour response data for ORs and ORNs from the cross-study normalised DoOR 2.0 database. We used the OR to uPN transform described in Olsen & Wilson [S10] to estimate uPN responses and then calculated their Gower distances, a method chosen to deal with large amounts of missing data. **B** Segregating odour spaces based on a literature review: most prominently using PubChem, the DoOR database, literature summaries from Huoviala *et al.* [S11] and Mansourian & Stensmyr [S12] as well as other recent work [S8, S12–S16]. Example strong ligands for glomeruli are given and in places their occurrence in nature (where this is clear and non-repetitive). Glomeruli are coloured by putative valences (appetitive green , aversive red , black unknown, based on estimates from the literature [S15], purple complex/dimorphic, orange disputed). **C** Hierarchical clustering (Ward's method, arbitrary height cut off for colouring at 0.3) of odour response data from Ca2+ responses in the dendrites of *Drosophila*, uniglomerular GH146 positive PNs [S17], after min-max normalisation and calculating their cosine distances.

Supplemental References

S1. Ito, M., Masuda, N., Shinomiya, K., Endo, K. & Ito, K. Systematic analysis of neural projections reveals clonal composition of the Drosophila brain. *Curr. Biol.* **23,** 644–655 (Apr. 2013).

S2. Wong, D. C., Lovick, J. K., Ngo, K. T., Borisuthirattana, W., Omoto, J. J. & Hartenstein, V. Postembryonic lineages of the Drosophila brain: II. Identification of lineage projection patterns based on MARCM clones. *Dev. Biol.* **384,** 258–289 (Dec. 2013).

S3. Lovick, J. K., Ngo, K. T., Omoto, J. J., Wong, D. C., Nguyen, J. D. & Hartenstein, V. Postembryonic lineages of the Drosophila brain: I. Development of the lineage-associated fiber tracts. *Dev. Biol.* **384,** 228–257 (Dec. 2013).

S4. Yu, H.-H., Awasaki, T., Schroeder, M. D., Long, F., Yang, J. S., He, Y., Ding, P., Kao, J.-C., Wu, G. Y.-Y., Peng, H., *et al.* Clonal Development and Organization of the Adult Drosophila Central Brain. *Curr. Biol.* **23** (Mar. 2013).

S5. Münch, D. & Galizia, C. G. DoOR 2.0–Comprehensive Mapping of Drosophila melanogaster Odorant Responses. *Sci. Rep.* **6,** 21841 (Feb. 2016).

S6. Schneider-Mizell, C. M., Gerhard, S., Longair, M., Kazimiers, T., Li, F., Zwart, M. F., Champion, A., Midgley, F. M., Fetter, R. D., Saalfeld, S., *et al.* Quantitative neuroanatomy for connectomics in Drosophila. *Elife* **5** (Mar. 2016).

S7. Frechter, S., Bates, A. S., Tootoonian, S., Dolan, M.-J., Manton, J., Jamasb, A. R., Kohl, J., Bock, D. & Jefferis, G. Functional and anatomical specificity in a higher olfactory centre. *eLife* **8** (eds VijayRaghavan, K. & Grunwald Kadow, I. C.) e44590. ISSN: 2050-084X (May 2019).

S8. Jeanne, J. M., Fişek, M. & Wilson, R. I. The Organization of Projections from Olfactory Glomeruli onto Higher-Order Neurons. *Neuron* **98,** 1198–1213.e6 (June 2018).

S9. Caron, S. J. C., Ruta, V., Abbott, L. F. & Axel, R. Random convergence of olfactory inputs in the Drosophila mushroom body. *Nature* **497,** 113–117 (May 2013).

S10. Olsen, S. R. & Wilson, R. I. Lateral presynaptic inhibition mediates gain control in an olfactory circuit. *Nature* **452,** 956–960 (Apr. 2008).

S11. Huoviala, P., Dolan, M.-J., Love, F. M., Frechter, S., Roberts, R. J., Mitrevica, Z., Schlegel, P., Bates, A. S., Aso, Y., Rodrigues, T., *et al.* Neural circuit basis of aversive odour processing in Drosophila from sensory input to descending output. *bioRxiv* (2018).

S12. Mansourian, S. & Stensmyr, M. C. The chemical ecology of the fly. *Curr. Opin. Neurobiol.* **34,** 95–102 (Oct. 2015).

S13. Dweck, H. K. M., Ebrahim, S. A. M., Thoma, M., Mohamed, A. A. M., Keesey, I. W., Trona, F., Lavista-Llanos, S., Svatoš, A., Sachse, S., Knaden, M., *et al.* Pheromones mediating copulation and attraction in Drosophila. *Proc. Natl. Acad. Sci. U. S. A.* **112,** E2829–35 (2015).

S14. Mohamed, A. A. M., Retzke, T., Das Chakraborty, S., Fabian, B., Hansson, B. S., Knaden, M. & Sachse, S. Odor mixtures of opposing valence unveil inter-glomerular crosstalk in the Drosophila antennal lobe. *Nat. Commun.* **10,** 1201 (Mar. 2019).

S15. Badel, L., Ohta, K., Tsuchimoto, Y. & Kazama, H. Decoding of Context-Dependent Olfactory Behavior in Drosophila. *Neuron* **91,** 155–167 (July 2016).

S16. Galizia, C. G., Münch, D., Strauch, M., Nissler, A. & Ma, S. Integrating heterogeneous odor response data into a common response model: A DoOR to the complete olfactome. *Chem. Senses* **35,** 551–563 (Sept. 2010).

S17. Badel, L., Ohta, K., Tsuchimoto, Y. & Kazama, H. Decoding of Context-Dependent Olfactory Behavior in Drosophila. *Neuron* **91,** 155–167 (2016).
