# Supplementary figures and images for: Complete Connectomic Reconstruction of Olfactory Projection Neurons in the Fly Brain
Source: Curr Biol. 2020 Aug 17;30(16):3183–3199.e6. doi: 10.1016/j.cub.2020.06.042 (PMC7443706; doi:10.1016/j.cub.2020.06.042)

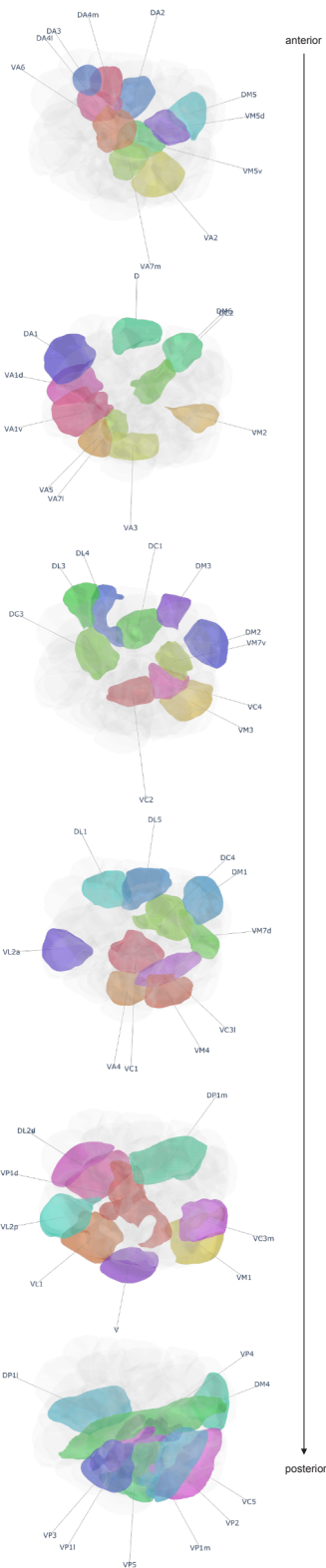

Supplement: Data S2 — Antennal Lobe Glomerulus Atlas, Related to Figure 1 Zip file containing a static (PDF) and an interactive 3D (HTML; open in web browser, click on items in the legend to show/hide objects) map of the antennal lobe glomeruli. [file mmc3.zip › glomeruli_atlas_static.pdf]
