# Supplementary material for: Complete Connectomic Reconstruction of Olfactory Projection Neurons in the Fly Brain
Source: Curr Biol. 2020 Aug 17;30(16):3183–3199.e6. doi: 10.1016/j.cub.2020.06.042 (PMC7443706; doi:10.1016/j.cub.2020.06.042)
Supplement: Document S2. Article plus Supplemental Information [file mmc8.pdf]

Current Biology

Article

# Complete Connectomic Reconstruction of Olfactory Projection Neurons in the Fly Brain

## Graphical Abstract

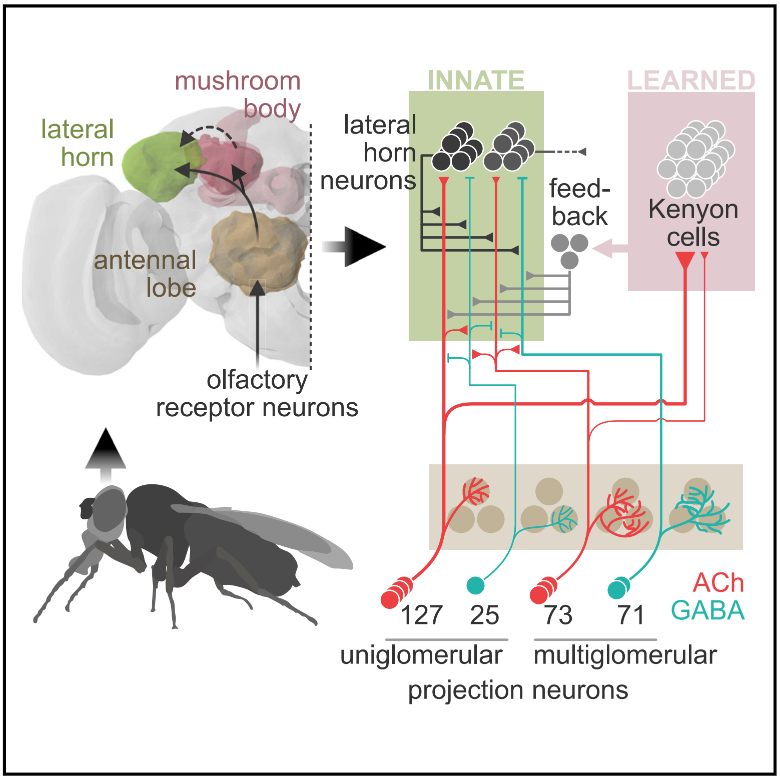


## Authors

Alexander S. Bates, Philipp Schlegel, Ruairi J.V. Roberts, ..., Davi D. Bock, Marta Costa, Gregory S.X.E. Jefferis

## Correspondence

jefferis@mrc-lmb.cam.ac.uk

## In Brief

Bates, Schlegel et al. report the first complete part list of second-order neurons in an adult arthropod olfactory system. They show that these neurons are stereotyped across hemispheres and reveal their hierarchical interconnectivity and how they connect to third-order targets and memory-related neurons.

## Highlights

- First complete parts list for second-order neurons of an adult olfactory system
- Quantification of left-right stereotypy in cell and synapse number
- Axo-axonic connections form hierarchical communities in the lateral horn
- Local and memory-related feedback target projection neuron axons

Bates et al., 2020, Current Biology *30*, 3183–3199
August 17, 2020 © 2020 MRC Laboratory of Molecular Biology.
Published by Elsevier Inc.
https://doi.org/10.1016/j.cub.2020.06.042

CellPress

Current Biology

CellPress
OPEN ACCESS

Article

# Complete Connectomic Reconstruction of Olfactory Projection Neurons in the Fly Brain

Alexander S. Bates,[1,7] Philipp Schlegel,[1,2,7] Ruairi J.V. Roberts,[2] Nikolas Drummond,[2] Imaan F.M. Tamimi,[2] Robert Turnbull,[2] Xincheng Zhao,[2,3] Elizabeth C. Marin,[2] Patricia D. Popovici,[1] Serene Dhawan,[2] Arian Jamasb,[2] Alexandre Javier,[2] Laia Serratosa Capdevila,[2] Feng Li,[4] Gerald M. Rubin,[4] Scott Waddell,[5] Davi D. Bock,[6] Marta Costa,[2] and Gregory S.X.E. Jefferis[1,2,8,*]

[1]Neurobiology Division, MRC Laboratory of Molecular Biology, Cambridge CB2 0QH, UK
[2]Department of Zoology, University of Cambridge, Cambridge CB2 3EJ, UK
[3]Department of Entomology, College of Plant Protection, Henan Agricultural University, Zhengzhou 450002, China
[4]Janelia Research Campus, Howard Hughes Medical Institute, Ashburn, VA 20147, USA
[5]Centre for Neural Circuits and Behaviour, The University of Oxford, Oxford OX1 3SR, UK
[6]Department of Neurological Sciences, Larner College of Medicine, University of Vermont, VT 05405, USA
[7]These authors contributed equally
[8]Lead Contact
*Correspondence: jefferis@mrc-lmb.cam.ac.uk
https://doi.org/10.1016/j.cub.2020.06.042

## SUMMARY

Nervous systems contain sensory neurons, local neurons, projection neurons, and motor neurons. To understand how these building blocks form whole circuits, we must distil these broad classes into neuronal cell types and describe their network connectivity. Using an electron micrograph dataset for an entire *Drosophila melanogaster* brain, we reconstruct the first complete inventory of olfactory projections connecting the antennal lobe, the insect analog of the mammalian olfactory bulb, to higher-order brain regions in an adult animal brain. We then connect this inventory to extant data in the literature, providing synaptic-resolution “holotypes” both for heavily investigated and previously unknown cell types. Projection neurons are approximately twice as numerous as reported by light level studies; cell types are stereotyped, but not identical, in cell and synapse numbers between brain hemispheres. The lateral horn, the insect analog of the mammalian cortical amygdala, is the main target for this olfactory information and has been shown to guide innate behavior. Here, we find new connectivity motifs, including axo-axonic connectivity between projection neurons, feedback, and lateral inhibition of these axons by a large population of neurons, and the convergence of different inputs, including non-olfactory inputs and memory-related feedback onto third-order olfactory neurons. These features are less prominent in the mushroom body calyx, the insect analog of the mammalian piriform cortex and a center for associative memory. Our work provides a complete neuroanatomical platform for future studies of the adult *Drosophila* olfactory system.

## INTRODUCTION

We now have a good mechanistic understanding of the principles of information processing in the primary olfactory center where first- and second-order neurons meet; this logic appears very similar between insects and mammals [1, 2]. However, even in the intensively studied vinegar fly, *Drosophila melanogaster*, much less is known about what happens in olfactory circuits beyond the second layer [3, 4]. In the insect brain, olfactory information is first received in the antennal lobe (AL; analogous to the mammalian olfactory bulb). Here, the axons of first-order olfactory receptor neurons (ORNs) ramify in 51 distinct olfactory glomeruli, a figure we finalize in the present work. Each glomerulus is targeted by a specific ORN type, defined by its odorant receptor(s). Besides the olfactory glomeruli, there are seven thermo- and hygrosensory glomeruli in the ventral-posterior (VP) part of the AL [5]. The AL implements local computations, including divisive normalization via lateral inhibition, before projection neurons (PNs; analogous to vertebrate mitral and tufted cells) carry information to higher brain regions (Figure 1A) [2]. PNs of the same cell type display broadly similar odor tuning across animals (e.g., [6–9]). Olfactory PNs target multiple brain regions including the well-studied mushroom body (MB), which appears analogous to the mammalian piriform cortex as both areas subserve associative olfactory memory [10–13] (Figure 1A). There has also been recent progress in understanding functional features of higher-order insect olfactory circuits outside of the MB [8, 14–20]. However, we lack a circuit-level framework of cell types and connections for the olfactory system to help contextualize these results. We now provide a full inventory of olfactory inputs to higher brain centers and use this to examine third-order olfactory processing. This work creates a neuroinformatic scaffold that can integrate diverse data from other studies.


This is an open access article under the CC BY license (http://creativecommons.org/licenses/by/4.0/).

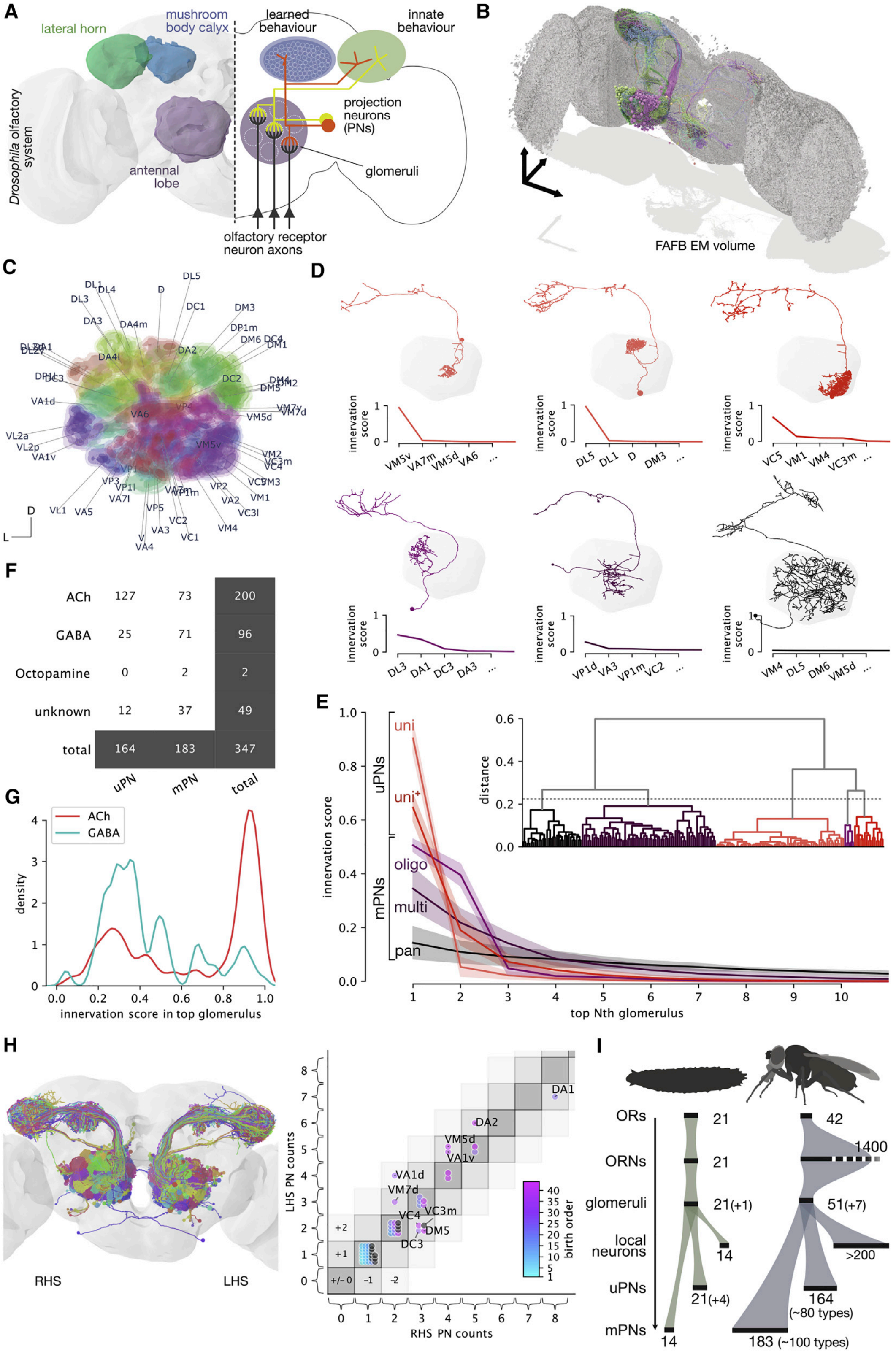


(legend on next page)

## RESULTS

Electron microscopy (EM) is the only means to resolve fine neurites (<200 nm), synaptic vesicles (~40 nm), and synaptic clefts (~20 nm). Using the recent full adult fly brain (FAFB) EM dataset [21], we created a full inventory of antennal lobe (AL) projection neurons (PNs).

### A Full Account of Olfactory Projection Neurons Connecting Second- and Third-Order Brain Areas

Previous PN surveys were based on sparse labeling and always incomplete. In FAFB, we found 347 PNs with dendrites in the right AL. We reconstructed their axons to completion and dendrites to identification (Figure 1B), cross-referencing each PN with extant data (developmental hemilineages, putative neurotransmitter expression) [22, 23] (Figure S1A; Data S3; see STAR Methods) [24–28]. Amidst conflicting accounts, we finalize the number of AL glomeruli at 51 olfactory and seven non-olfactory [5].

PN dendrites range from innervating a single glomerulus to almost the entire AL (Figure 1D; Video S1). To quantitatively describe their innervation patterns, we generated a probabilistic model of the AL and calculated innervation scores for all PN-glomerulus pairs (Figure 1C; see STAR Methods and Data S1 and S2 for atlas and meshes). By convention, PNs are classified as either uni- or multiglomerular [21–23, 29, 30]. These broad classes facilitate comparisons, though obscure the diversity in PN morphology. For classification, we generated "traces" for each PN, sorting innervation scores by strength ($1^{st}$, $2^{nd}$, ..., $58^{th}$) and clustered PNs using these traces (Figure 1E). Two superclusters intuitively correspond with uni/oligo- and multiglomerular PNs, but the fine structure suggests more subtypes, which we tentatively identify as uni-, uni+, oligo-, multi-, and pan-glomerular. For comparison with prior studies, we broadly group PNs into uniglomerular (uPNs: uni, uni+ clusters) and multiglomerular (mPNs: oligo, multi, pan). Recent work using this EM volume identified 114 uPNs [15, 21]. We update this count to 164 uniglomerular and 183 multiglomerular PNs; 2–3 times more than previously reported [23, 29, 31].

Cholinergic PNs appear, on average, to be sparser than GABAergic PNs (Figure 1G); indeed, most uPNs are cholinergic while mPNs are split between cholinergic and GABAergic (Figure 1F). We define 80 uPN types based on hemilineage, glomerulus innervated, and the antennal lobe tract through which they project (Figure S1A). To assess numerical variability, we reconstructed all members of 58 uPN types on the left-hand side to identification. Left versus right numbers vary by 1.1 ± 0.3 SD for 10/58 types (Figure 1H).

### The Synaptic Organization of Olfactory Projection Neurons

We next turned to third-order olfactory neuropils. We annotated all chemical synapses on PN axons (Figure 2A). The lateral horn (LH) and the MB calyx (CA) are the main targets, both by number of incoming PNs and synapses per neuropil (Figures 2B, 2C, and S2B). However, the LH receives almost all the AL's feedforward inhibition, though GABAergic synapses make up only ~15% of its olfactory input. PN axons in the LH (but not CA) are heavily modulated: for example, the DP1m uPN receives 1,236 synaptic inputs on its LH axon but only 50 in the CA. Postsynapse (input) density along axons in the LH was rather constant: 0.19 ± 0.008 (SEM) per micron of cable (Figure 2D). Presynapse density was also consistent but differed between cholinergic uPNs with greater than twice the presynapse density of GABAergic PNs (1.14 ± 0.008 versus 0.47 ± 0.02 per micron). Insect synapses are polyadic [32], i.e., each presynaptic site has multiple associated postsynaptic sites (Figure 2A). The number of postsynaptic sites at individual presynapses can vary 10-fold, but the average value was the same for uPNs versus mPNs and cholinergic versus GABAergic neurons (approximately 12 ± 0.1 SEM) (Figure 2E).

Next, we calculated an anatomical transfer function, relating each olfactory channel to the number of PN synapses in higher brain areas. For multiglomerular PNs, we weighted inputs by the proportion of dendritic arbor per glomerulus (see STAR Methods). There is a strong positive correlation (0.78 Pearson's r, $p < 0.001$) between glomerulus volume and the number of axonal output synapses of their cognate PNs (Figures 2F, S2D, and S2E). Second, glomeruli supply varying ratios of cholinergic:GABAergic and uPN:mPN drive to higher brain neuropils (Figure 2G). For example, DM1 has 3,898 feed-forward presynapses: 86% associated with cholinergic uPNs, 9% cholinergic mPNs, and just 2% with GABAergic PNs. In contrast, VL1's budget of 2,404 output synapses is divided 30% cholinergic uPNs, 36% cholinergic mPNs, and 26% GABAergic PNs. Therefore, while feedforward uniglomerular cholinergic excitation is the dominant output of the AL, the profile for individual glomeruli can differ widely.

To assess stereotypy in synaptic output, we compared right and left hemisphere PNs. We cross-matched PN types consisting of a single neuron (DL4, DA4l, DL1, DA4m) or multiple sisters (DA1, DA2, VA1d) and reconstructed left-side axons to synaptic

**Figure 1. Second-Order Olfactory Projection Neurons**

(A) Schematic of fly olfactory system.

(B) Second-order olfactory projection neurons (PNs) reconstructed from a serial section transmission electron microscopy volume of an entire fly brain (FAFB). Arrows represent 100 microns.

(C) Probabilistic model of antennal lobe (AL) glomeruli.

(D) Exemplary sparse to broad PNs (top left → bottom right). Traces show innervation scores derived from AL model (top four glomeruli shown).

(E) Innervation traces of PNs clustered by pairwise euclidean distance (dendrogram). Clusters of different PN classes of PNs were grouped into uniglomerular (uni, uni+) and multiglomerular (oligo, multi, pan) PNs. Envelopes represent SD.

(F) PN counts by class and neurotransmitter.

(G) Top innervation score by neurotransmitter. Most cholinergic PNs are sparse; most GABAergic PNs are broad.

(H) Comparison of right- (RHS) versus left-hand-side (LHS) cell counts for 58/80 uPN types.

(I) Scaling of olfactory system from larva to adult *D. melanogaster*.

See also Figure S1; Data S1, S2, and S3; Videos S1 and S2.

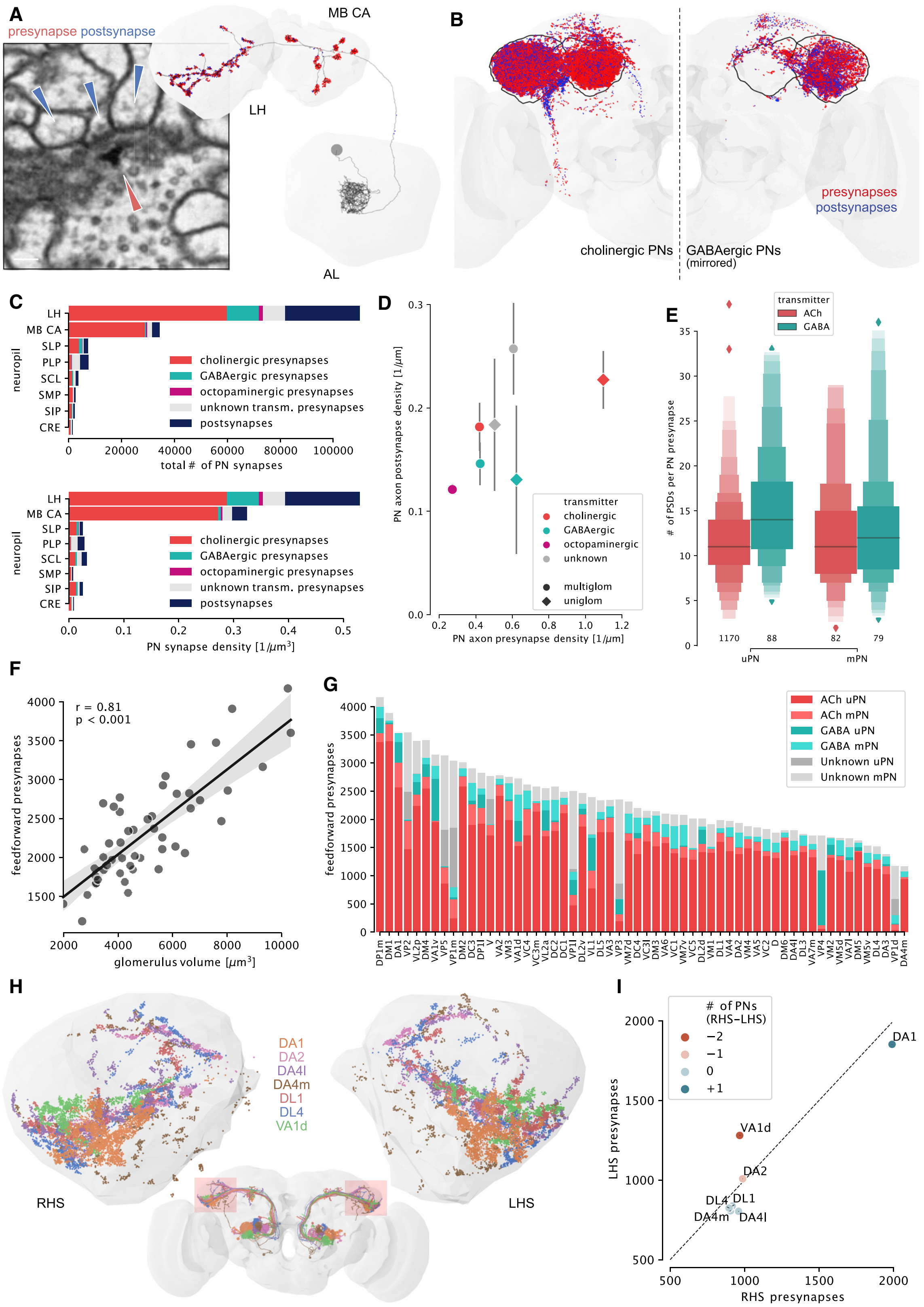


(legend on next page)

CellPress
OPEN ACCESS

completion (Figure 2H). The total number of pre- and postsynapses differ between the left and right homologs by only 12% (Figures 2I and S2F).

### Axo-axonic Network between Olfactory Projection Neurons

Unexpectedly, axo-axonic connections between PN axons are common in the LH, but rare in the CA and other higher brain regions (Figures 3A and 3B). This axo-axonic input derives predominantly from cholinergic PNs (Figure 3D), but cholinergic PNs receive less PN→PN input than GABAergic PNs (Figure 3C). In this PN→PN axo-axonic network, 26% of PNs are strongly connected (weighted degree $\geq$ 50), and except for 3 disconnected PNs (0.8%), the network is fully connected (Figure S3A). We find strong connections between sister PNs (e.g., DA1) but also across types, many of which are not reciprocal (i.e., axon A connects to B, but B does not connect to A). A low reciprocity is indicative of a hierarchical network [34], and indeed, only 7.4% of edge weights are reciprocated—compared with 29.7% in a proximity-based null model (see "Presynapses within Range" in STAR Methods).

We used out-degree (the sum of all outgoing edge weights) and local reaching centrality (LRC; the weighted fraction of other neurons reachable via outgoing connections) [35] to explore the position of neurons within this network (Figure 3E). A high out-degree suggests a neuron exerts control over its direct partners. LRC measures how far that control can spread; low LRC suggests local influence, and high LRC suggests global influence. Both metrics gave similar results across PN classes (Figure S3B), but individual PNs stand out: cholinergic uPNs for DM1, VL1 (both food-related), DA4m, DC4 (both aversive), VP2 (heating), a GABAergic VP3 (cooling) uPN, and a few mPNs have above average out-degrees/LRCs (Figures 3F and S3C). For example, the cholinergic DM1 uPN has a high out-degree but only an average LRC and therefore appears to be a local hub that connects strongly onto a number of other food-related PNs like DM4 (Figure 3G). The network exhibits complex connectivity motifs such as feedback inhibition between the excitatory and inhibitory VL1 uPNs (Figures 3G and 3H).

The LH can be roughly compartmentalized according to biological valence [8, 36–39]. To explore whether a similar organization exists within the PN→PN network, we applied a high-fidelity community detection algorithm [40]. This produced 13 communities including all but the three completely disconnected PNs (Figures 3I, 3J, S3D, and S3E). We then assigned odor scenes (Figure S7) to each community based on the glomeruli innervated by their PNs (Figure 3I) [41, 42]. For example, PNs in community 2 mainly innervate pheromone-responsive glomeruli DA1, DL3, DC3, VA1v, matching a previously described pheromonal LH compartment (Figure S3E) [36, 37]. Other communities show equally distinct fingerprints, suggesting that odor scene specialization may govern the topology of the network.

### Feedforward Olfactory Input to Neurons of the Lateral Horn

To understand how third-order neurons receive olfactory input, we selected a morphologically diverse set of 82 lateral horn neurons (LHNs) for complete synaptic reconstruction; 56 LH output neurons (LHONs) and 26 local neurons (LHLNs) (see STAR Methods). We defined morphological cell types (matching to known cell types when possible) (Data S4). All examined LHLN cell types are either GABAergic or glutamatergic [14] and probably inhibitory [43, 44]. We contrast these with a set of 15 MB Kenyon cells (KCs) [21]. Most insect neurons have a cell body fiber that enters the neuropil and bifurcates, one branch forming its dendrite and the other its axon (Figure S4A). All 82 LHNs have two major arbors that can be separated into putative dendrite and axon based on the density of output synapses (Figures 4A and 4B). Significantly, this is also true for LHLNs, which have more presynapses per micron of axonal (0.22 ± 0.07 SD) than dendritic cable (0.08 ± 0.03 SD) and almost double the density of synaptic inputs on their dendrites (0.39 ± 0.09 SD) compared with axons. Conversely, some LHON axons experience heavy, and some very little, modulation—e.g., 85–111 synaptic inputs on LHPD2a1 but only 10–57 on LHPV5a1. Axonal arbors are smaller but thicker than dendrites (Figures S4C and S4D). Interestingly, the dendritic compartment is consistently closer to the soma than the axonal compartment (Figure 4B). Together, this suggests that the two compartments are functionally and developmentally distinct, even in local neurons.

Each LHN combines excitatory drives from multiple glomeruli via different uPN cell types, which account for 34% ± 14% SD of their postsynaptic budget. PNs account for twice as much of the input onto KCs: 79% ± 6% SD. Each LHN samples sparsely, though not quite as sparsely as KCs, from the 80 uPN types. LHLN axons are sparser than the dendrites of the same cells (Figure 4C), providing further support for two functionally distinct compartments. LHONs sample from 6.9 ± 4.9 SD cholinergic uPNs, LHLNs from 6.0 ± 3.5 SD (1.5% of total synaptic input cut-off, Figure S4B). This is similar to reports from light

**Figure 2. Third-Order Targets of Olfactory Feedforward Drive**

(A) Axonal pre- (outputs, red arrow) and postsynaptic (inputs, blue arrows) sites for exemplar PN.

(B) Spatial distribution of pre- and postsynapses for cholinergic and GABAergic PNs (see Figure S2A for Octopamine and unknown transmitter).

(C) Synapse number (top) and density (bottom) by target neuropil. LH and CA are main targets of feedforward olfactory drive. Most modulatory input onto PN axons occurs in LH.

(D) Synapse densities along axon. Presynapse densities vary strongly: uniglomerular, cholinergic PNs have more than twice the density of GABAergic PNs. Error bars represent SEM.

(E) Number of postsynaptic densities (PSDs) per LH synapse (polyadicity). Letter-value plot encodes distribution of data by showing additional quantiles [33]; black line represents mean; numbers below plots are synapses sampled.

(F) Glomerulus volume strongly correlates with the number of feedforward PN synapses (Pearson).

(G) Feedforward synapse budget per glomerulus. Glomeruli differ widely in the proportions of excitation to inhibition and uni- to multiglomerular synapse budgets.

(H) Synapses of left (LHS) and right (RHS) homologs of 7 uPN types in the LH.

(I) Comparison of left and right presynapse counts between uPN types in (H). Synapse numbers are consistent even in case of mismatches in number of cells. See also Figure S2; Data S1.

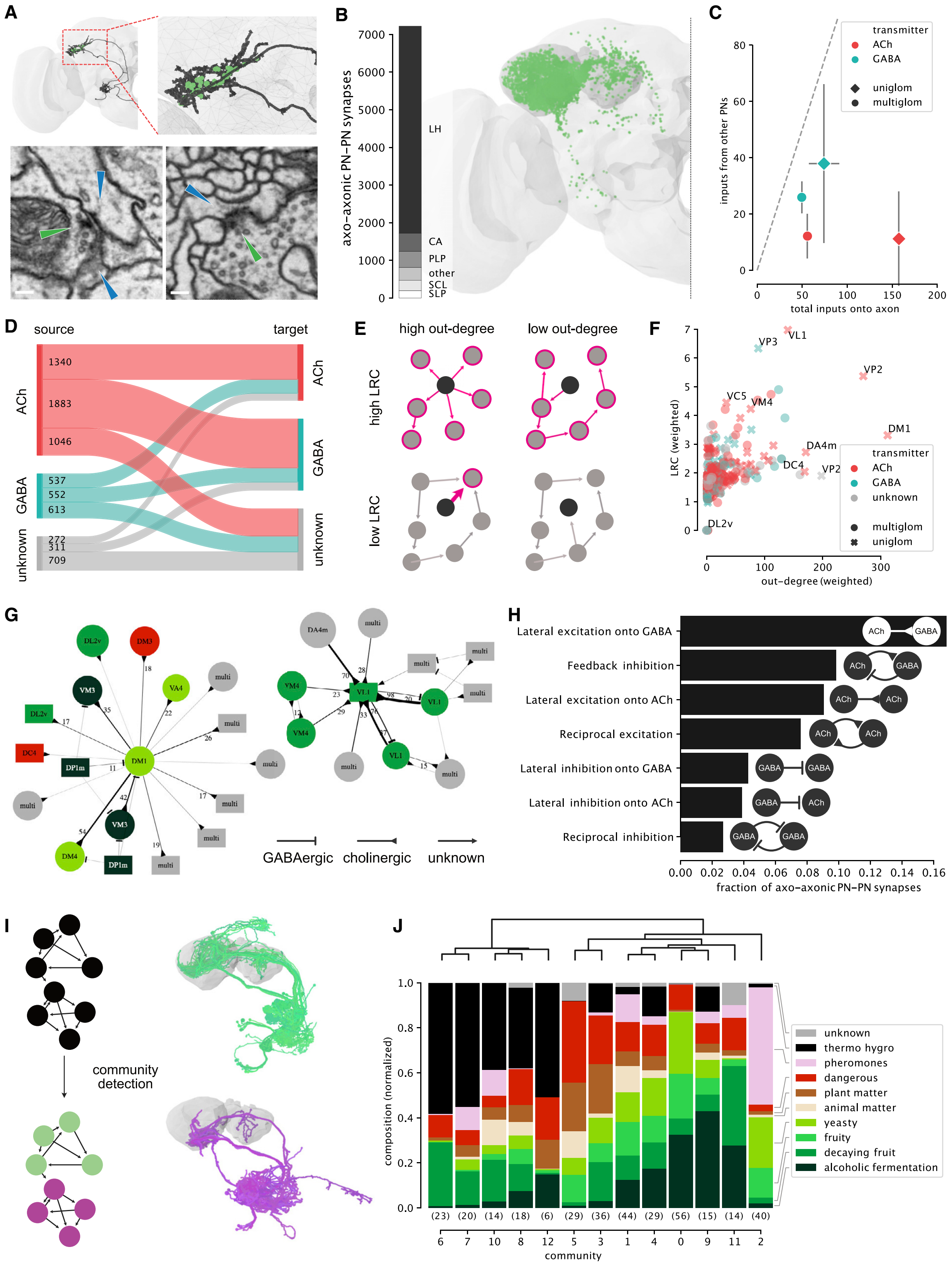


*(legend on next page)*

microscopy data for both KCs (6.0 ± 1.2 SD) and LHNs (6.2) [18, 46]. On average, there is comparatively little GABAergic or mPN input (Figure 4D), although LHLN axons receive more feedforward inhibition than LHLN or LHON dendrites. To test whether neurons preferentially target other neurons by class, we predicted connectivity between PNs and 82 third-order neurons using three models: overlap score, potential contacts, and presynapses within range ("near synapses") (Figure S4E; see STAR Methods). A simple overlap score correlates well with the observed connectivity, especially for axo-dendritic PN→LHON connections (Figure S4E). However, non-synaptic connectivity prediction methods tend to over-predict the strength of unitary connections between pairs of neurons (Figure S4F). Predicting connectivity based on whether a potential upstream neuron has output synapses near a potential downstream neuron, shows that the proportion to which these unitary connections are "filled" differs depending on both neuron class and compartment (Figure 4F). For example, a larger fraction of these "potential synapses" are formed between uPN axons and KC dendrites than for uPN→LHON connections, which in turn are more frequent than axo-axonic uPN→LHLN or uPN→cholinergic PN connections. Axo-axonic predictions often differed substantially from predictions (Figure 4E), in part because many strong axo-axonic connections are unidirectional (Figure S5E).

## Neuron Class- and Compartment-Specific Upstream Connectomes

Only 21% of KC dendritic input comes from neurons other than olfactory PNs. For LHNs, this fraction is much larger: 60% of inputs to LHONs and 47% to LHLNs in the LH (Figure 4D). Similarly, 72% of inputs onto PN axons in the LH are not from fellow olfactory PNs. What could be supplying this synaptic input? We reconstructed upstream partners of all LH PN axons and 6 LHNs (3 LHONs, 3 LHLNs) to identify them (Figure S5A; 79% of postsynapses sampled). There is a long-tailed distribution of connection strengths, with many weak partners, but this differs between compartments and neuron classes (Figure 5A). For example, ~80% of upstream partner neurons of LHON and LHLN dendrites are "weak" (individually <1% of synaptic inputs). For their axons, this distribution is shifted (LHLNs 45%, LHONs 35%, respectively), becoming more similar to PN axons (19%). LHLNs account for about a third of all input: 37% for LHLN dendrites, 26% for LHON dendrites, and 30% for PN axons (Figures 5B and S5B). This number is much smaller (4.5%) for LHLN axons, suggesting that LHLN axons might avoid one another. Both LHONs and LHLNs receive input from many neurons, a mean of 196 and 120, respectively. However, only 9% of LHLN→LHLN and 20% of LHLN→LHON unitary connections are "strong" (>1% of its input budget, a mean of 5 strongly connected LHLNs). In contrast, 86% of LHLN→PN unitary connections are strong (a mean of 7 strongly connected LHLNs). Unexpectedly, LHON dendrites provide considerable input to other LHON dendrites (13%), LHLN dendrites (14%) and axons (14%), and PN axons (5%). This suggests they are more than simple outlets of the LH but participate in local computations. Interestingly, the fraction of direct GABAergic mPN input is generally small (<3%), except for LHLN axons, where it accounts for 18%.

Distinct fingerprints between neuron classes can be recovered using dimensionality reduction techniques on neurons' upstream connectivity vectors (Figures 5C and 5D). Clustering PNs by these vectors weakly correlates with a purely morphological clustering, using Baker's Gamma Index [47, 48] (Figure 5E, see legend). High morphological similarity often means very similar connectivity, but even small morphological differences can result in large differences in input (Figure 5F, note many lines crossing in small sets in tanglegram). Modulation of LHON axons may depend on behavioral context: LHPD2a1#1 and LHPV6a1#1 receive input from food-related PNs and respond to food-related odors [8, 20], and at least LHPD2a1 is also modulated by olfactory context and memory [14, 15, 50]. Both receive a large amount of memory-related axo-axonic "feedback" (34% compared with 27% axo-dendritic) in addition to input from other LHON axons (Figure S5B). In contrast, LHAV1a1#1, a neuron involved in innate repulsion from bacterial odors [17], is not as highly modulated: no axo-axonic memory-related feedback, minimal (0.2%) memory-related axo-dendritic input, and little LHON-LHON axo-axonic connectivity (6%). Furthermore, two bilateral, GABAergic LHPV12a1 [8, 14] interneurons innervate a wide range of PN axons (5% of all PN axonic input comes from just these two cells) but also receive input from PN axons (Figures 6D and 6E). These neurons may act to normalize activity between the CA and LH across hemispheres. Interestingly, 12% of PN axon input comes from a new class of LH input neurons, putative mechanosensory neurons. Another new class, "LH centrifugal neurons", accounts for 5% (as well as 11% of LHON and 2% of LHLN synaptic inputs), and some of these unitary connections are high, e.g., the

**Figure 3. Axo-axonic Communities between Olfactory Projection Neurons**

(A) Axo-axonic synapses between olfactory PNs occur onto large backbones (lower left) and small twigs (lower right). Presynapses and corresponding postsynapses labeled with green and blue arrows, respectively; scale bar, 100 nm.

(B) The majority of axo-axonic connections between PNs occur in the lateral horn.

(C) Fraction of axonic inputs from other PNs. GABAergic PNs receive a higher fraction of inputs from other PNs than cholinergic PNs. Error bars represent SEM.

(D) Flow chart visualizing axo-axonic connections between PNs by neurotransmitter.

(E) Network analyses: nodes with high out-degree (many strong outgoing connections) are hubs in the network, nodes with high local reaching centrality (LRC) can potentially reach many nodes in the network (see STAR Methods for details).

(F) Hierarchy analysis within the PN→PN network using LRC and out-degree. A few, mostly cholinergic uniglomerular PNs, represent major hubs in the network.

(G) Graphs of two exemplary subnetworks around DM1 (left) and GABAergic VL1 (right) uPNs. Colors correspond to odor scene as in (J); numbers indicate synapses per unitary connection.

(H) Quantification of motifs in axo-axonic PN-PN network. Arrowheads as in (G).

(I) Community detection splits PN-PN network into 13 spatially overlapping communities (two examples shown).

(J) Composition of communities by odor scene shows distinct preferences. Number of neurons per community given in brackets.

See also Figure S3 and S7; Data S1, S2, and S3.

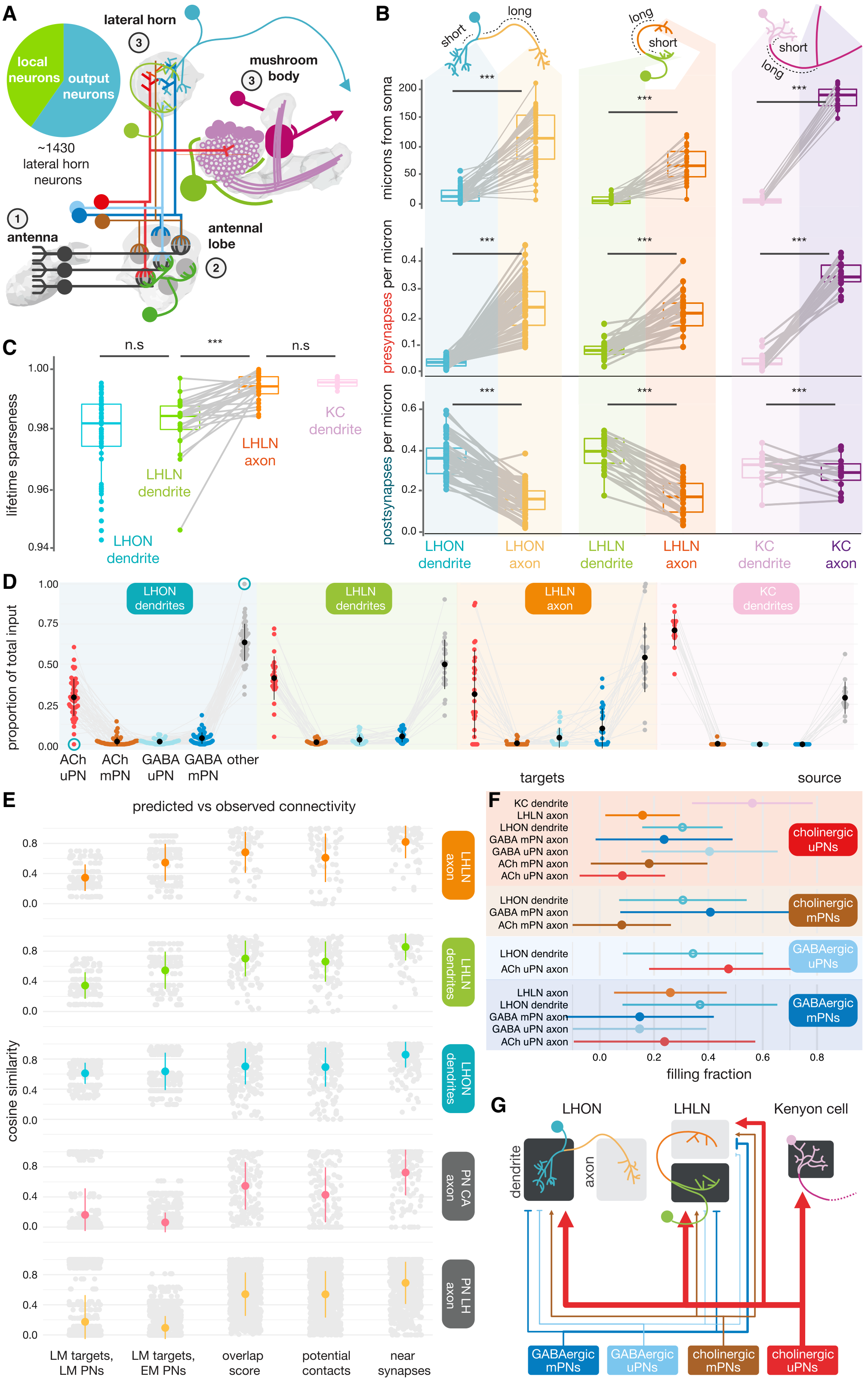


**Figure 4. Feedforward Olfactory Input to Innate Center Neurons**

(A) Neuroanatomical schematic of the first three layers of the insect olfactory system. Pie chart shows proportion of local and output neurons among an estimated ~1,400 LHNs.

(legend continued on next page)

DM1 uPN axon receives 196 connections from a single LH centrifugal neuron.

### Third-Order Mechanosensory Neurons Receive Innate and Learned Olfactory Information

The LH also receives non-olfactory input, including mechanosensory from wedge projection neurons (WEDPNs 1–4) [14] (Figures S6A and S6C). We identified four additional WEDPN types (see STAR Methods), fully reconstructing one neuron per type (Figure S6C). Unusually, some WEDPNs (types 2–4, 6, and 7) have a second dendrite located in the LH (Figures S6A–S6C). Comprehensive upstream sampling of exemplar WEDPNs 1–4 revealed feedforward mechanosensory input from the contralateral antennal mechanosensory and motor center (AMMC) and input from local neurons of the wedge and surrounding neuropils. The LH dendrite of WEDPNs 2–4 receives major input from a class of glutamatergic LHLN, LHPV4a, and a MB output neuron (MBON) class, MBONs-$\alpha'3$ (Figures S6B and S6E). LHPV4a are classifiers for amine odors [8], whose optogenetic activation induces aversive behavior [14]. MBONs-$\alpha'3$ mediate aversive memory recall [11, 51] and detection of novel odors [52]. Interestingly, MBONs-$\alpha'3$'s cognate dopaminergic neuron, PPL1 $\alpha'3$ [10], receives GABAergic innervation from WEDPNs as well as cholinergic drive from a related MBON subtype (MBONs-$\alpha'3$ap). This motif may be key to their novelty-detection role [52, 53] (Figure S6E).

### Higher-Order Brain Areas Feedback onto the Lateral Horn

Up to ~25% (average 2.5%) of inputs onto uPN axons is from 10 novel "LH centrifugal neurons" (Figures 6A and 6B), which feedback to the LH, and often also the CA, from the LH's target regions [8, 14, 18]. Like MBONs, centrifugal neurons are cytoplasmically large, synapse-dense, and presumably metabolically expensive cells. They primarily appear as one neuron per cell type per hemisphere. In the LH and CA, they receive input from olfactory PNs and also target olfactory PN axons (Figures 6E and 6F). Select MBONs provide strong synaptic input onto centrifugal neurons' dendrites (Figure 6E), in particular cholinergic MBONs that mediate aversive memory recall and naive attraction [11, 52], as well as the glutamatergic MBON-$\gamma5\beta'2$a [54], which has the opposite profile and targets LHCENT1 with 618 synapses. These connections can be reciprocal. We only have one synaptically complete MBON axon type [15], and for it, we observed a strong reciprocal connection with LHCENT1: 126 synapses onto and 61 from the MBON axon (Figure 6F). We also observed a single neuron of similarly gross morphology but opposite polarization to LHCENT neurons, that innervates the MB lobes (LHMB1). The only other cell known to input the MB lobes from the LH is PPL1 $\alpha'3$ [10].

## DISCUSSION

### Numerical Stereotypy among Olfactory Projection Neurons

We leveraged a whole *D. melanogaster* brain EM volume [21] to obtain a full inventory of the 347 PNs that relay olfactory information to higher brain centers (Figures 1C and S1A). Because PNs are morphologically stereotyped [36, 55, 56], our reconstructions serve as holotypes for identifying these cells across datasets [57]. Genetic driver lines can label different numbers of cells across animals [31], but it is unclear whether this represents actual differences in cell numbers or merely variations in expression. A recent study showed a difference in numbers for a single uPN type between the left and right hemisphere within one animal [58]. We now tested numerical stereotypy of 58 uPN types across hemispheres and found a variability of up to 50% (2 RHS versus 4 LHS VA1d uPNs) in ~17% of cases (Figure 1G). When neuron numbers vary, synapse numbers may change to maintain consistent connection strengths. Tobin et al. [58] reported such compensation for ORN→PN synapses within the DM6 glomerulus. We found for DA1 and DA2 uPNs (LHS/RHS count: 7/8 and 6/5, respectively) (Figure 1G) that the total number of axonal synapses is almost identical across hemispheres (Figures 2H, 2I, and S2F). This compensation is somewhat surprising because connectivity is much more distributed in the LH. Compensation may have limits as VA1d uPNs, which are twice as numerous on one side, showed only partial compensation (1.3× rather than 1× synapses).

### Scaling Up an Olfactory System

The adult olfactory system faces more complex challenges than its larval equivalent. The number of AL glomeruli increases from 21 to 51 (+7), a ~2.5× increase proportional to the number of olfactory receptors [59]. However, the numbers of AL local neurons, uPNs, and mPNs scale supralinear with glomerulus number, increasing by factors of 5, 6, and 14, respectively (Figure 1I) [2, 23, 59, 60]. The number of mPN cell types rises from 14 to an estimated 100 in the adult. This supralinear increase in

(B) Paired boxplots comparing key metrics between for third-order olfactory neurons' dendrites versus their axons. Paired Student's t test, ***p ≤ 0.001.
(C) Lifetime sparseness [45] for third-order olfactory neuron compartments sampling the uPN population (~80 cell types). Lifetime sparseness is a measure of selectivity that is calculated separately for each single neuron across all patterns (i.e., weighted input connectivity vector), the higher the value the sparser the neuron. Paired Student's t test, ***p ≤ 0.001.
(D) Proportion of synaptic inputs supplied by feedforward olfactory drive from the AL (pooled by neuron class and compartment). Blue circle highlights LHPV5g1#1, a putative LHON that receives little second-order olfactory input.
(E) Cosine similarity between different methods of synaptic prediction and observed synaptic connectivity in the EM dataset (see STAR Methods). Only olfactory PN connectivity onto different classes of downstream target (facets) are considered. LM = registered light microscopy data [8, 30]. Cosine similarity examines whether neurons connect to the same targets in similar proportions (it is the cosine of the angle between connectivity vectors in a multi-dimensional space).
(F) The weighted mean (± SD) of the filling fraction between two neurons (no connections predicted by looking for nearby presynapses divided by observed connections), for olfactory PN connections onto different classes of downstream targets. Only results statistically significantly different from the PN→LHON dendrite filling fraction (open circle, blue) are shown (weighted Student's t test). Number of near synapses used as the weights.
(G) Schematic of feedforward input from the AL to the LH and CA.
See also Figure S4; Data S1 and S4.

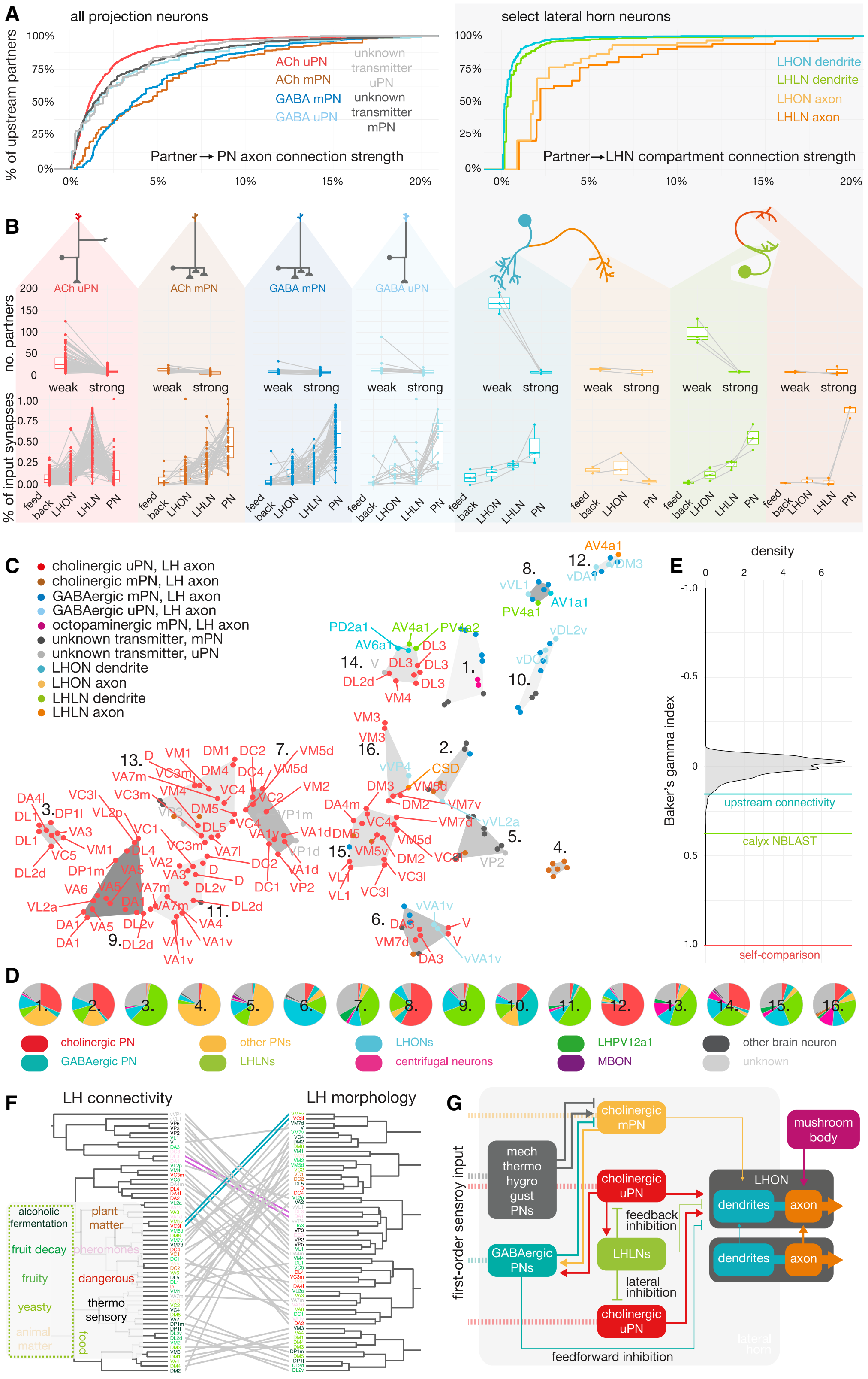


(legend on next page)

CellPress
OPEN ACCESS

PN diversity may reflect an exponentially larger number of glomerular combinations to decode. Curiously, although mPNs are numerous, they receive less ORN input than uPNs and make far fewer output synapses in higher areas and so may individually be less influential (Figures 2D, 2E, and S1E).

## Axo-axonic Connectivity in the LH

The LH is comparable with the mammalian cortical amygdala in containing third-order olfactory neurons necessary for categorizing odors and some innate olfactory responses [8, 14, 15, 61–63]. We report a hierarchical network between PN axons within the LH (Figure 3). These axo-axonic connections (which are confined to the LH) may help the LH categorize odors without affecting the ability of the MB to discriminate odors (Figure 3B). The biophysical impact of axo-axonic connections is unclear—they can have non-intuitive effects depending on the timing of action potentials in connected partners [64–67]. Nevertheless, their presence strongly suggests that odor channels directly influence one another. Perhaps they circumvent the need to produce additional metabolically expensive local neurons. For example, the DM1 uniglomerular PN is upstream of multiple, food-odor-tuned PNs, such as DM4 (Figure 3G). Some food-related odor channels may directly facilitate the action of others. A new study observed a community of PNs in the MB CA, a food-related "associational fovea", densely sampled by a population of KCs [68]. This community is almost identical to the food-related, axo-axonic community we observe in the LH (note that both studies use the same EM data). While the association between food-responsive PN types is conserved between LH and CA, the functional interaction is different.

## Connectivity Depends on Neuron Class and Compartment

We fully reconstructed 82/∼1,400 LHNs. Based on this sample, LHNs have statistically separable dendrites and axons even though both can make and receive chemical synapses (Figures 4B and S4D). Dendrites were consistently located closer to the soma than axons. LHONs had a large number of synaptic inputs on their dendrites (mean 600), while LHLNs had a mean of 545 inputs across both arbors. LHNs have hundreds of upstream partners with varying degrees of unitary connection strength; surprisingly, ∼80% of LHN inputs are "weak" connections (Figure 5B). The ratio of potential and actual synapses differed markedly across connection types. This suggests class-specific developmental processes may create the feedforward connectivity motifs from the AL to the LH (Figures 7, 4F, and 4G), perhaps by using different gains for Peter's rule [69]. For example, individual local neurons can have different input-output relationships with different PN axons, implementing asymmetric lateral inhibition (Figures S5E and S5F). In one specific example, the LHAV4a1#1 LHLN indirectly connects two adjacent PN arbors: DM1, which responds to apple cider vinegar, and DP1m, which responds to acids [70]. The two PN axons overlap but do not connect directly instead interacting via DM1 PN→LHLN→DP1m PN inhibition (Figure S5E). Such motifs may enable the fly to balance the attractive nature of food sources against repulsive levels of acidity.

## Structural Differences between Olfactory Neuropils

We sought to deduce some general features of circuit organization, leveraging our new results and comparing four olfactory areas (AL, LH, MB CA, and lobes) each containing input, output, and local neurons (Figure 7A). Olfactory PN input is modulated by LH local neurons, making uPN→LHLN→uPN lateral inhibition a prominent feature of the LH, but not the CA (Figure S5C). This is comparable with the situation in the AL and MB lobes where input neurons (ORNs and KCs) receive a significant amount of input from feedback and local neurons [58, 73]—though the numbers of feedback and local neurons are very different (Figure 7A). LHONs receive a significantly smaller fraction of feedforward input from olfactory PNs than MB KCs (46% versus >80%). We wonder whether there is an analogy with antennal lobe PNs: uniglomerular PNs receive strong feedforward input from ORNs [58, 73], whereas multiglomerular PNs receive more diverse input (Figure S1E). In the AL, LH and MB lobes the dendrites of output neurons can talk back to input neurons (Figures 5B, 5C, and S1E), while output neurons of the CA (KCs) appear to have few dendritic presynapses (Figure S4D). Among these four neuropils, the LH is unique in that its input neurons (PNs) strongly interact with one another in a hierarchical manner, and that it receives GABAergic as well as cholinergic input (Figure 3).

**Figure 5. Upstream Connectivity of PNs in the LH and for Select LH Neurons**

(A) Left, empirical cumulative density plots for the unitary connection strengths of all upstream partners to downstream PN axons and 6 exemplary LHNs.

(B) Top, number of weak (<1% of target's synapses accounted for by an upstream partner) and strong (≥1%) synaptic partners. Bottom, proportion of neurons' total inputs coming from feedback neurons, LHONs, LHLNs, and olfactory PNs, where "feedback" refers to input from MBONs and LH centrifugal neurons. Split by class and compartment (colored facets).

(C) A t-SNE plot based on synaptic budget vectors for each target neuron, where each value in the vector is the normalized number of synapses supplied by a different upstream neuron class. Grey polygons correspond to a K-means clustering, k = 16.

(D) Pie charts show how the input budget of selected clusters in (C) is spent.

(E) Density plot of hierarchical clustering similarity scores (Baker's gamma index, [47, 48]) between a hierarchical clustering of NBLAST scores [49] for PN axons in the LH and 10,000 random permutations of this clustering (red line, self-comparison). Baker's gamma index between this morphological clustering and an upstream connectivity clustering shown in blue (0.21, one-sided p value < 0.005), and a comparison with a NBLAST cluster of calycal terminals in green (0.24, one-sided p value < 0.005). To calculate this similarity index, the maximum number of clusters for which each pair of neurons falls into the same grouping is computed. A Spearman correlation is then calculated between two sets of these numbers, one for each hierarchical clustering.

(F) Tanglegram comparing hierarchical clustering based on upstream connectivity similarity (cosine similarity between vectors for synaptic connectivity) and morphological similarity (NBLAST). Label colors indicate the coarse odor scene in which uPNs are likely to respond [42]. If a PN falls into more than one odor scene, only one color is chosen. Colored lines indicate groups of neurons clustered together in both dendrograms.

(G) Schematic of the connectivity motifs found in the LH.

See also Figure S5; Data S1 and S4.

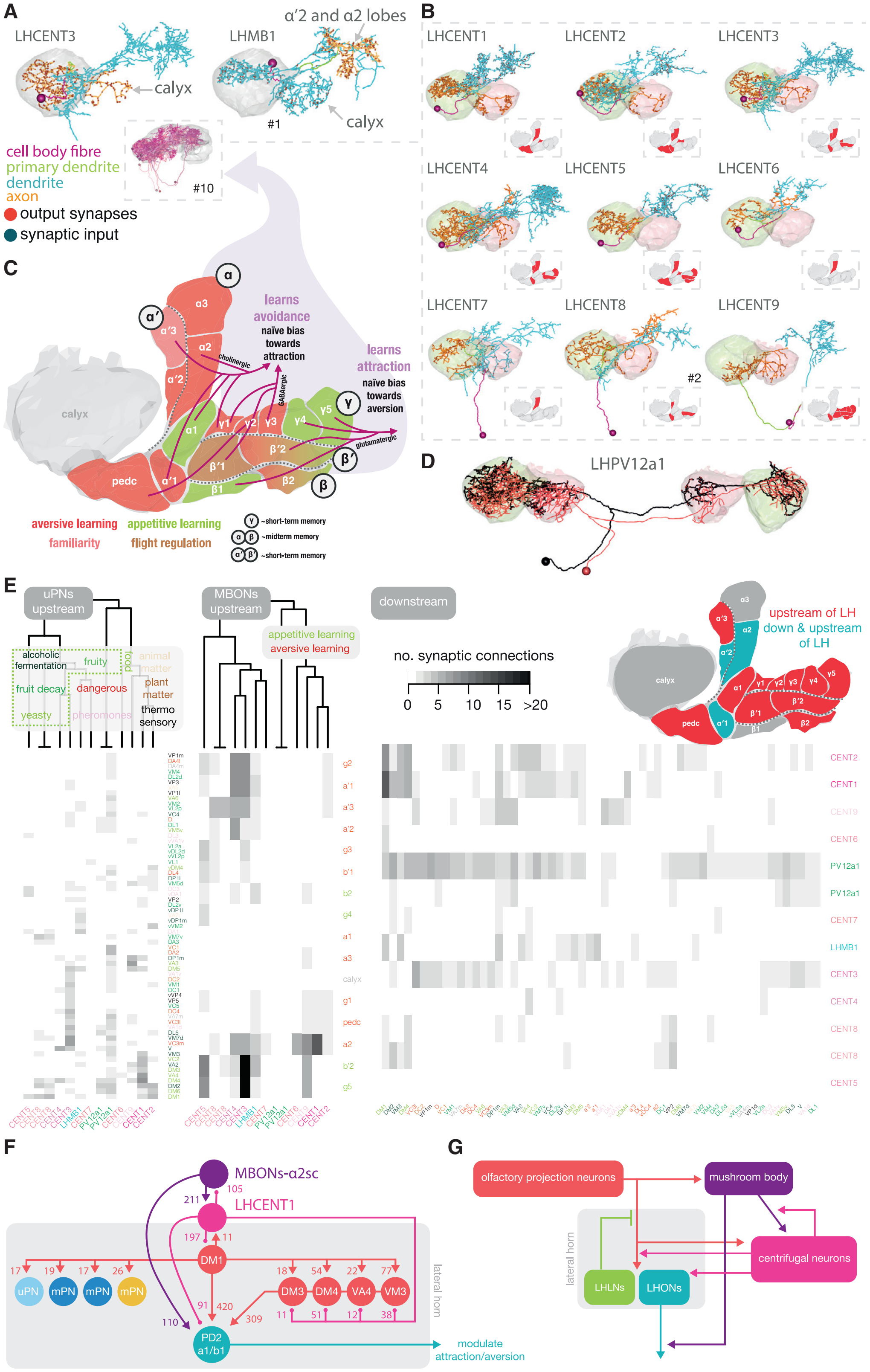


**Figure 6. Feedback Memory Input to the LH**

(A) Left, an example of an LH centrifugal neuron. Inset, all neurons of the class shown in purple hues. Right, LHMB1, a single LHON that targets the MB lobes.

(B) 10 LH centrifugal neurons form 9 cell types. Insets depict the MB compartments, whose MBONs innervate LHCENT neurons.

(legend continued on next page)

## Mechanosensory-Olfactory Integration Occurs in the LH

Wind perception is crucial for navigation, including anemotaxis (following odors upwind) or flight in general. The LH receives projections from the wedge, a third-order mechanosensory region [8, 14, 74–76] (Figure S6A). Genetic activation of WEDPN1 (R37E08-GAL4) generates increased wing-flick motion and differential wing-angling [77] (Figure S6C). In the LH, WEDPNs receive both diverse olfactory input and connections from the MB via MBON-$\alpha'$3 (Figure S6E), whose activation makes flies enter an "attentive" state that suppresses other behaviors [52]. We hypothesize that WEDPNs, as major downstream targets, may mediate this effect and suggest that WEDPN modulation by olfactory inputs could also focus attention on certain cues, e.g., polyamines [78], which are of high ethological significance. WEDPNs also target the reinforcing dopaminergic neuron for the MB $\alpha$'3 compartment. We propose that this circuit (Figure S6E) may mediate context-dependent changes in attention or familiarization-learning rates.

## Memory-Related Control of "Innate" Center Neurons

Previously described neurons that directly connect the MB and LH include MB-C1, MBONs-$\alpha$sc, MBONs-$\alpha'$3, PPL1 $\alpha'$3, and LHPV12a1 [8, 10, 14, 15, 21, 51]. Here, we discovered a major new class that we termed "LH centrifugal neurons" (Figure 6B); their dendrites receive input from diverse MBONs (Figure 6E), while their axons target a range of neurons in the LH, including PN axons (Figure 7). This may be one way in which MBONs promote specific behaviors after learning. Because the axons of centrifugal neurons target PN axons in the LH, we suggest that they allow the MB to modulate the gain of pathways through the LH rather than directly activating particular behavioral programm. For example, LHCENT1 is downstream of MBON-$\alpha$2sc and strongly synapses onto the axon of the DM1 uPN (hub of the food-odor PN community) (Figure S6F). Intriguingly, we recently showed that LHON cell types (PD2a1/b1), which receive convergent input from DM1 and MBON-$\alpha$2sc, are required for aversive memory retrieval [15]. This new LHCENT1 circuit likely enables the MB to change the gain of the attraction-promoting DM1 pathway through the LH. Clearly the MB alpha lobe and LH are strongly interconnected. This may give memory hierarchical control over "innate" circuits and could be used either to suppress those circuits, favoring a learned behavioral response in some contexts, or to recruit "innate" circuitry in order to produce desired, learned outcomes. Furthermore, the DM1 PN→PN community could reciprocally influence the MB alpha lobe via the LHMB1 neuron. This connection could also allow food-odors to act as a training signal or slow the rate of aversive learning paired with food odors, making it harder to override critical positive instincts. Taken together with other recent work [14, 15, 79, 80], we conclude that the MB and LH are much more extensively interconnected than previously appreciated. Functional studies of this interconnectivity will be crucial to understanding the organization of learned and innate behaviors.

## STAR★METHODS

Detailed methods are provided in the online version of this paper and include the following:

- KEY RESOURCES TABLE
- RESOURCE AVAILABILITY
  - Lead Contact
  - Materials Availability
  - Data and Code Availability
- METHOD DETAILS
  - Neuronal Reconstruction
  - Bridging EM and light-level Data
  - PN Types and Putative Neurotransmitters
  - Connectivity Modeling
  - Compartmentalising Neurons
  - Morphological Analysis
  - Antennal Lobe Model
  - Network Analysis
- QUANTIFICATION AND STATISTICAL ANALYSIS

## SUPPLEMENTAL INFORMATION

Supplemental Information can be found online at https://doi.org/10.1016/j.cub.2020.06.042.

## ACKNOWLEDGMENTS

This work was supported by a Wellcome Trust Collaborative Award (203261/Z/16/Z) to G.S.X.E.J, D.B., G.M.R., and S.W.; an ERC Consolidator grant (649111) and core support from the MRC (MC-U105188491) to G.S.X.E.J.; a Boehringer Ingelheim Fonds PhD Fellowship and a Herchel Smith Studentship to A.S.B.; and a China Scholarship Council award (no. 201908410128) to X.Z. We thank Volker Hartenstein for his help in identifying the lineages that contain lateral horn neurons. Neuronal reconstruction for this project took place in a collaborative CATMAID environment in which 27 labs are participating to build connectomes for specific circuits. Therefore, work done for published (Dolan et al. [14, 15], Zheng et al. [21], Sayin et al. [80], and Coates et al. [72]) and ongoing projects has proved useful to us. We thank Fiona Love, Billy Morris, Amelia Edmondson-Stait, Kimberly Meechan, Jawaid Ali, Clement Hallou, Mahmoud Elbahnasawi, Lucia Kmecova, Paavo Huoviala, Zane Mitrevica, Nadiya Sharifi, István Taisz, Irene Varela, Najla Masoodpanah, Melissa Ryan, Peter Gibb, Kaylynn Coates, Shanice Bailey, Quinn Vanderbeck, Jason Polsky, Joseph Hsu, Benjamin Gorko, Jacob Ratliff, Michael Lingelbach, Aidan Smith, Amalia Braun, Adam Heath, Corey Fisher, Kathi Eichler, Ben Koppenhaver, Jeremy Johnson, Przemyslaw Jarzebowski, Remy Tabano, Claire Managan, Johann Schor, Katie Stevens, Adam John, Shada Alghailani, Lindsey Tagg,

(C) Diagram of the MB lobes, colored by putative valence [3, 11].
(D) FAFB EM reconstruction of a large bilateral interneuron, LHPV12a1 [14] that connects both CAs and LHs (black) and its light-level match from a recent split-GAL4 screen (red).
(E) Heatmaps showing the connectivity of uPNs→MB-LH neurons (left), MBONs→MB-LH neurons (middle) by compartment and, conversely MB-LH neurons→uPN axons and MB-LH neurons→MBON dendrites (right). PN and MB compartment names colored by odor scene and valence respectively. If a PN falls into more than one odor scene, only one color is chosen.
(F) Example with synaptic weights of how memory can control or modulate lateral horn neurons known to be important for aversive memory recall [14] (arrows = acetylcholine; circles = transmitter unknown).
(G) Schematic depicting how MBONs interact with LHNs via LH centrifugal neurons. Arrows can be of inhibitory, excitatory or unknown sign.
See also Figures S6 and S7; Data S1 and S4.

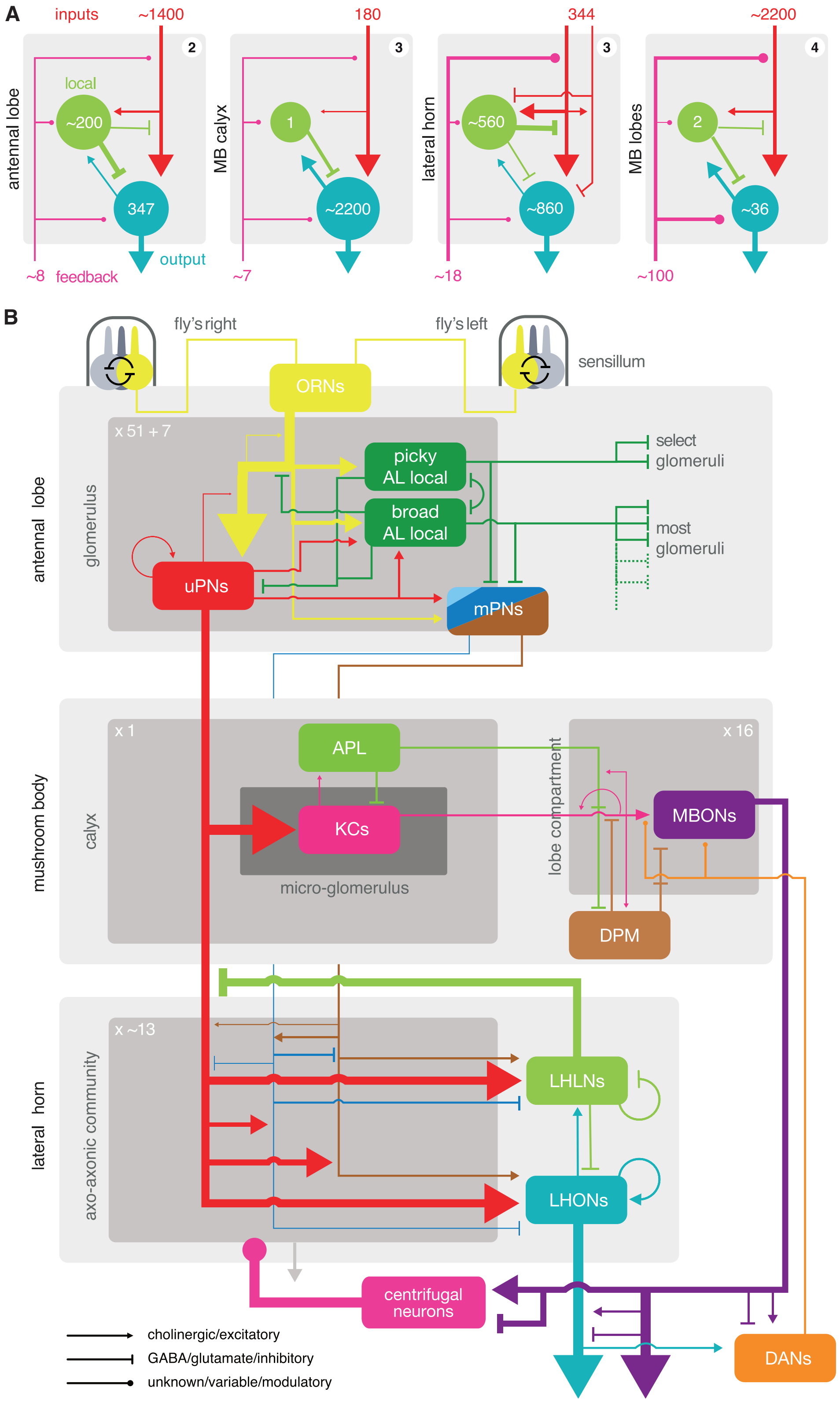


**Figure 7. Summary Wiring Diagrams of the *D. melanogaster* Olfactory System**

(A) Basic wiring diagrams for four major olfactory neuropils in the fly brain, in terms of input, local, output, and feedback neurons. Line widths approximate the strength of neuron-to-neuron connections.

*(legend continued on next page)*

CellPress
OPEN ACCESS

Philipp Ranft, Bailey Harrison, Steven Calle, Shahrozia Imtiaz, Gabrielle Allred, Markus Pleijzier, Ala Haddad, Emily Tenshaw, Prescott Martin, Austin Warner, Sarah Farris, Georgia Dempsey, Raquel Frances, Dana Galili, Addy Adesina, Levi Helmick, Scott Lauritzen, Farzaan Salman, Andrew Dacks, Kabas Abou Jahjah, Konrad Heinz, Kai Liang, and Maria Theiss for contributing ~29% of the novel neuronal cable used in this study manuscript. We thank Tom Kazimiers for maintaining CATMAID software and servers. We thank Peter Li for sharing his automatic segmentation, and Eric Perlman for importing these into CATMAID.

## AUTHOR CONTRIBUTIONS

A.S.B., P.S., R.J.V.R., N.D., I.F.M.T., X.Z., E.C.M., P.D.P., S.D., A. Jamasb, A. Javier, L.S.C., F.L., M.C., D.D.B., and G.S.X.E.J. reconstructed neurons and their connectivity from the EM data. A.S.B., P.S., E.C.M., M.C., and G.S.X.E.J. conceived this study and analyzed the data. G.S.X.E.J., D.D.B., G.M.R., and S.W. supervised this study and obtained funding. A.S.B., P.S., M.C., and G.S.X.E.J. wrote the manuscript with input from all authors.

## DECLARATION OF INTERESTS

The authors declare no competing interests.

Received: January 20, 2020
Revised: May 7, 2020
Accepted: June 12, 2020
Published: July 2, 2020

## REFERENCES

1. Masse, N.Y., Turner, G.C., and Jefferis, G.S.X.E. (2009). Olfactory information processing in Drosophila. Curr. Biol. *19*, R700–R713.
2. Wilson, R.I. (2013). Early olfactory processing in Drosophila: mechanisms and principles. Annu. Rev. Neurosci. *36*, 217–241.
3. Amin, H., and Lin, A.C. (2019). Neuronal mechanisms underlying innate and learned olfactory processing in Drosophila. Curr. Opin. Insect Sci. *36*, 9–17.
4. Groschner, L.N., and Miesenböck, G. (2019). Mechanisms of Sensory Discrimination: Insights from *Drosophila* Olfaction. Annu. Rev. Biophys. *48*, 209–229.
5. Marin, E.C., Roberts, R.J., Büld, L., Theiss, M., Pleijzier, M.W., Sarkissian, T., Laursen, W.J., Turnbull, R., Schlegel, P., Bates, A.S., et al. (2020). Connectomics analysis reveals first, second, and third order thermosensory and hygrosensory neurons in the adult Drosophila brain. Curr. Biol. *30*. Published online July 2, 2020. https://doi.org/10.1016/j.cub.2020.06.028.
6. Wilson, R.I., Turner, G.C., and Laurent, G. (2004). Transformation of olfactory representations in the Drosophila antennal lobe. Science *303*, 366–370.
7. Bhandawat, V., Olsen, S.R., Gouwens, N.W., Schlief, M.L., and Wilson, R.I. (2007). Sensory processing in the Drosophila antennal lobe increases reliability and separability of ensemble odor representations. Nat. Neurosci. *10*, 1474–1482.
8. Frechter, S., Bates, A.S., Tootoonian, S., Dolan, M.-J., Manton, J., Jamasb, A.R., Kohl, J., Bock, D., and Jefferis, G. (2019). Functional and anatomical specificity in a higher olfactory centre. eLife *8*, e44590.
9. Honegger, K.S., Smith, M.A.-Y., Churgin, M.A., Turner, G.C., and de Bivort, B.L. (2019). Idiosyncratic neural coding and neuromodulation of olfactory individuality in Drosophila. Proc. Natl. Acad. Sci. U.S.A. 201901623.
10. Aso, Y., Hattori, D., Yu, Y., Johnston, R.M., Iyer, N.A., Ngo, T.-T., Dionne, H., Abbott, L.F., Axel, R., Tanimoto, H., and Rubin, G.M. (2014). The neuronal architecture of the mushroom body provides a logic for associative learning. eLife *3*, e04577.
11. Aso, Y., Sitaraman, D., Ichinose, T., Kaun, K.R., Vogt, K., Belliart-Guérin, G., Plaçais, P.-Y., Robie, A.A., Yamagata, N., Schnaitmann, C., et al. (2014). Mushroom body output neurons encode valence and guide memory-based action selection in Drosophila. eLife *3*, e04580.
12. Choi, G.B., Stettler, D.D., Kallman, B.R., Bhaskar, S.T., Fleischmann, A., and Axel, R. (2011). Driving opposing behaviors with ensembles of piriform neurons. Cell *146*, 1004–1015.
13. Franks, K.M., Russo, M.J., Sosulski, D.L., Mulligan, A.A., Siegelbaum, S.A., and Axel, R. (2011). Recurrent circuitry dynamically shapes the activation of piriform cortex. Neuron *72*, 49–56.
14. Dolan, M.-J., Frechter, S., Bates, A.S., Dan, C., Huoviala, P., Roberts, R.J., Schlegel, P., Dhawan, S., Tabano, R., Dionne, H., et al. (2019). Neurogenetic dissection of the *Drosophila* lateral horn reveals major outputs, diverse behavioural functions, and interactions with the mushroom body. eLife *8*, e43079.
15. Dolan, M.-J., Belliart-Guérin, G., Bates, A.S., Frechter, S., Lampin-Saint-Amaux, A., Aso, Y., Roberts, R.J.V., Schlegel, P., Wong, A., Hammad, A., et al. (2018). Communication from Learned to Innate Olfactory Processing Centers Is Required for Memory Retrieval in Drosophila. Neuron *100*, 651–668.e8.
16. Varela, N., Gaspar, M., Dias, S., and Vasconcelos, M.L. (2019). Avoidance response to CO2 in the lateral horn. PLoS Biol. *17*, e2006749.
17. Huoviala, P., Dolan, M.-J., Love, F.M., Frechter, S., Roberts, R.J., Mitrevica, Z., Schlegel, P., Bates, A.S., Aso, Y., Rodrigues, T., et al. (2018). Neural circuit basis of aversive odour processing in Drosophila from sensory input to descending output. bioRxiv. https://doi.org/10.1101/394403.
18. Jeanne, J.M., Fişek, M., and Wilson, R.I. (2018). The Organization of Projections from Olfactory Glomeruli onto Higher-Order Neurons. Neuron *98*, 1198–1213.e6.
19. Jeanne, J.M., and Wilson, R.I. (2015). Convergence, Divergence, and Reconvergence in a Feedforward Network Improves Neural Speed and Accuracy. Neuron *88*, 1014–1026.
20. Fişek, M., and Wilson, R.I. (2014). Stereotyped connectivity and computations in higher-order olfactory neurons. Nat. Neurosci. *17*, 280–288.
21. Zheng, Z., Lauritzen, J.S., Perlman, E., Robinson, C.G., Nichols, M., Milkie, D., Torrens, O., Price, J., Fisher, C.B., Sharifi, N., et al. (2018). A Complete Electron Microscopy Volume of the Brain of Adult Drosophila melanogaster. Cell *174*, 730–743.e22.
22. Tanaka, N.K., Suzuki, E., Dye, L., Ejima, A., and Stopfer, M. (2012). Dye fills reveal additional olfactory tracts in the protocerebrum of wild-type Drosophila. J. Comp. Neurol. *520*, 4131–4140.
23. Tanaka, N.K., Endo, K., and Ito, K. (2012). Organization of antennal lobe-associated neurons in adult Drosophila melanogaster brain. J. Comp. Neurol. *520*, 4067–4130.
24. Lacin, H., Chen, H.-M., Long, X., Singer, R.H., Lee, T., and Truman, J.W. (2019). Neurotransmitter identity is acquired in a lineage-restricted manner in the Drosophila CNS. eLife *8*, e43701.
25. Ito, M., Masuda, N., Shinomiya, K., Endo, K., and Ito, K. (2013). Systematic analysis of neural projections reveals clonal composition of the Drosophila brain. Curr. Biol. *23*, 644–655.

(B) The overall layout of the olfactory system of *D. melanogaster*, including LH wiring uncovered by the present study. Note that connectivity as regards to LHNs is based on 82/~1,400 LHNs and so represents connection types we have observed, though others may exist. 1,400 ORNs, 2,200 KCs, ~200 AL LNs, 347 PNs, 35 MBONS, 10 MB LH Cent, 8 AL feedback (e.g., AL-MDBL), 100 DANs, 1 APL, 1 DPM, 560 LHLNs, and 830 LHONs [8, 10, 31, 46, 60, 71]. Feedback neurons to the AL have not previously been observed (except for the clearly unpolarized CSD neurons [72]); we found and partially reconstructed a further seven examples of neurons with dendrites in the superior protocerebrum and a putative axon in the AL (data not shown). Examined LHLNs have all proven GABAergic or glutamatergic and most LHONs have been shown to be cholinergic [14].

26. Wong, D.C., Lovick, J.K., Ngo, K.T., Borisuthirattana, W., Omoto, J.J., and Hartenstein, V. (2013). Postembryonic lineages of the Drosophila brain: II. Identification of lineage projection patterns based on MARCM clones. Dev. Biol. *384*, 258–289.

27. Lovick, J.K., Ngo, K.T., Omoto, J.J., Wong, D.C., Nguyen, J.D., and Hartenstein, V. (2013). Postembryonic lineages of the Drosophila brain: I. Development of the lineage-associated fiber tracts. Dev. Biol. *384*, 228–257.

28. Yu, H.-H., Awasaki, T., Schroeder, M.D., Long, F., Yang, J.S., He, Y., Ding, P., Kao, J.-C., Wu, G.Y.-Y., Peng, H., et al. (2013). Clonal development and organization of the adult Drosophila central brain. Curr. Biol. *23*, 633–643.

29. Yu, H.-H., Kao, C.-F., He, Y., Ding, P., Kao, J.-C., and Lee, T. (2010). A complete developmental sequence of a Drosophila neuronal lineage as revealed by twin-spot MARCM. PLoS Biol. *8*, e1000461.

30. Chiang, A.-S., Lin, C.-Y., Chuang, C.-C., Chang, H.-M., Hsieh, C.-H., Yeh, C.-W., Shih, C.-T., Wu, J.-J., Wang, G.-T., Chen, Y.-C., et al. (2011). Three-dimensional reconstruction of brain-wide wiring networks in Drosophila at single-cell resolution. Curr. Biol. *21*, 1–11.

31. Grabe, V., Baschwitz, A., Dweck, H.K.M., Lavista-Llanos, S., Hansson, B.S., and Sachse, S. (2016). Elucidating the Neuronal Architecture of Olfactory Glomeruli in the Drosophila Antennal Lobe. Cell Rep. *16*, 3401–3413.

32. Meinertzhagen, I.A., and O'Neil, S.D. (1991). Synaptic organization of columnar elements in the lamina of the wild type in Drosophila melanogaster. J. Comp. Neurol. *305*, 232–263.

33. Heike, H., Wickham, H., and Kafadar, K. (2017). Letter-Value Plots: Boxplots for Large Data. J. Comput. Graph. Stat. *26*, 469–477.

34. Squartini, T., Picciolo, F., Ruzzenenti, F., and Garlaschelli, D. (2013). Reciprocity of weighted networks. Sci Rep. *3*, 2729.

35. Mones, E., Vicsek, L., and Vicsek, T. (2012). Hierarchy measure for complex networks. PLoS ONE *7*, e33799.

36. Jefferis, G.S.X.E., Potter, C.J., Chan, A.M., Marin, E.C., Rohlfing, T., Maurer, C.R., Jr., and Luo, L. (2007). Comprehensive maps of Drosophila higher olfactory centers: spatially segregated fruit and pheromone representation. Cell *128*, 1187–1203.

37. Bates, A.S., Manton, J.D., Jagannathan, S.R., Costa, M., Schlegel, P., Rohlfing, T., and Jefferis, G.S. (2020). The natverse, a versatile toolbox for combining and analysing neuroanatomical data. eLife *9*, e53350.

38. Strutz, A., Soelter, J., Baschwitz, A., Farhan, A., Grabe, V., Rybak, J., Knaden, M., Schmuker, M., Hansson, B.S., and Sachse, S. (2014). Decoding odor quality and intensity in the Drosophila brain. eLife *3*, e04147.

39. Tanaka, N.K., Awasaki, T., Shimada, T., and Ito, K. (2004). Integration of chemosensory pathways in the Drosophila second-order olfactory centers. Curr. Biol. *14*, 449–457.

40. Traag, V.A., Waltman, L., and van Eck, N.J. (2019). From Louvain to Leiden: guaranteeing well-connected communities. Sci. Rep. *9*, 5233.

41. Badel, L., Ohta, K., Tsuchimoto, Y., and Kazama, H. (2016). Decoding of Context-Dependent Olfactory Behavior in Drosophila. Neuron *91*, 155–167.

42. Mansourian, S., and Stensmyr, M.C. (2015). The chemical ecology of the fly. Curr. Opin. Neurobiol. *34*, 95–102.

43. Cully, D.F., Paress, P.S., Liu, K.K., Schaeffer, J.M., and Arena, J.P. (1996). Identification of a Drosophila melanogaster glutamate-gated chloride channel sensitive to the antiparasitic agent avermectin. J. Biol. Chem. *271*, 20187–20191.

44. Liu, W.W., and Wilson, R.I. (2013). Glutamate is an inhibitory neurotransmitter in the Drosophila olfactory system. Proc. Natl. Acad. Sci. USA *110*, 10294–10299.

45. Willmore, B., and Tolhurst, D.J. (2001). Characterizing the sparseness of neural codes. Network *12*, 255–270.

46. Caron, S.J.C., Ruta, V., Abbott, L.F., and Axel, R. (2013). Random convergence of olfactory inputs in the Drosophila mushroom body. Nature *497*, 113–117.

47. Baker, F.B. (1974). Stability of Two Hierarchical Grouping Techniques Case I: Sensitivity to Data Errors. J. Am. Stat. Assoc. *69*, 440–445.

48. Galili, T. (2015). dendextend: an R package for visualizing, adjusting and comparing trees of hierarchical clustering. Bioinformatics *31*, 3718–3720.

49. Costa, M., Manton, J.D., Ostrovsky, A.D., Prohaska, S., and Jefferis, G.S.X.E. (2016). NBLAST: Rapid, Sensitive Comparison of Neuronal Structure and Construction of Neuron Family Databases. Neuron *91*, 293–311.

50. Lerner, H., Rozenfeld, E., Rozenman, B., Huetteroth, W., and Parnas, M. (2020). Differential Role for a Defined Lateral Horn Neuron Subset in Naïve Odor Valence in Drosophila. Sci. Rep. *10*, 6147.

51. Séjourné, J., Plaçais, P.-Y., Aso, Y., Siwanowicz, I., Trannoy, S., Thoma, V., Tedjakumala, S.R., Rubin, G.M., Tchénio, P., Ito, K., et al. (2011). Mushroom body efferent neurons responsible for aversive olfactory memory retrieval in Drosophila. Nat. Neurosci. *14*, 903–910.

52. Hattori, D., Aso, Y., Swartz, K.J., Rubin, G.M., Abbott, L.F., and Axel, R. (2017). Representations of Novelty and Familiarity in a Mushroom Body Compartment. Cell *169*, 956–969.e17.

53. Cognigni, P., Felsenberg, J., and Waddell, S. (2018). Do the right thing: neural network mechanisms of memory formation, expression and update in Drosophila. Curr. Opin. Neurobiol. *49*, 51–58.

54. Owald, D., Felsenberg, J., Talbot, C.B., Das, G., Perisse, E., Huetteroth, W., and Waddell, S. (2015). Activity of defined mushroom body output neurons underlies learned olfactory behavior in Drosophila. Neuron *86*, 417–427.

55. Wong, A.M., Wang, J.W., and Axel, R. (2002). Spatial representation of the glomerular map in the Drosophila protocerebrum. Cell *109*, 229–241.

56. Marin, E.C., Jefferis, G.S.X.E., Komiyama, T., Zhu, H., and Luo, L. (2002). Representation of the glomerular olfactory map in the Drosophila brain. Cell *109*, 243–255.

57. Bates, A.S., Janssens, J., Jefferis, G.S., and Aerts, S. (2019). Neuronal cell types in the fly: single-cell anatomy meets single-cell genomics. Curr. Opin. Neurobiol. *56*, 125–134.

58. Tobin, W.F., Wilson, R.I., and Lee, W.A. (2017). Wiring variations that enable and constrain neural computation in a sensory microcircuit. eLife *6*, e24838.

59. Berck, M.E., Khandelwal, A., Claus, L., Hernandez-Nunez, L., Si, G., Tabone, C.J., Li, F., Truman, J.W., Fetter, R.D., Louis, M., et al. (2016). The wiring diagram of a glomerular olfactory system. eLife *5*, e14859.

60. Chou, Y.-H., Spletter, M.L., Yaksi, E., Leong, J.C.S., Wilson, R.I., and Luo, L. (2010). Diversity and wiring variability of olfactory local interneurons in the Drosophila antennal lobe. Nat. Neurosci. *13*, 439–449.

61. Root, C.M., Denny, C.A., Hen, R., and Axel, R. (2014). The participation of cortical amygdala in innate, odour-driven behaviour. Nature *515*, 269–273.

62. Iurilli, G., and Datta, S.R. (2017). Population Coding in an Innately Relevant Olfactory Area. Neuron *93*, 1180–1197.e7.

63. Heimbeck, G., Bugnon, V., Gendre, N., Keller, A., and Stocker, R.F. (2001). A central neural circuit for experience-independent olfactory and courtship behavior in Drosophila melanogaster. Proc. Natl. Acad. Sci. USA *98*, 15336–15341.

64. Burrows, M., and Laurent, G. (1993). Synaptic potentials in the central terminals of locust proprioceptive afferents generated by other afferents from the same sense organ. J. Neurosci. *13*, 808–819.

65. Burrows, M., and Matheson, T. (1994). A presynaptic gain control mechanism among sensory neurons of a locust leg proprioceptor. J. Neurosci. *14*, 272–282.

66. Haag, J., and Borst, A. (2004). Neural mechanism underlying complex receptive field properties of motion-sensitive interneurons. Nat. Neurosci. *7*, 628–634.

67. Cuntz, H., Haag, J., Forstner, F., Segev, I., and Borst, A. (2007). Robust coding of flow-field parameters by axo-axonal gap junctions between fly visual interneurons. Proc. Natl. Acad. Sci. USA *104*, 10229–10233.

68. Zheng, Z., Li, F., Fisher, C., Ali, I.J., Sharifi, N., Calle-Schuler, S., Hsu, J., Masoodpanah, N., Kmecova, L., Kazimiers, T., et al. (2020). Structured sampling of olfactory input by the fly mushroom body. bioRxiv. https://doi.org/10.1101/2020.04.17.047167.

69. Rees, C.L., Moradi, K., and Ascoli, G.A. (2017). Weighing the Evidence in Peters' Rule: Does Neuronal Morphology Predict Connectivity? Trends Neurosci. *40*, 63–71.

70. Ai, M., Min, S., Grosjean, Y., Leblanc, C., Bell, R., Benton, R., and Suh, G.S.B. (2010). Acid sensing by the Drosophila olfactory system. Nature *468*, 691–695.

71. Lin, A.C., Bygrave, A.M., de Calignon, A., Lee, T., and Miesenböck, G. (2014). Sparse, decorrelated odor coding in the mushroom body enhances learned odor discrimination. Nat. Neurosci. *17*, 559–568.

72. Coates, K.E., Calle-Schuler, S.A., Helmick, L.M., Knotts, V.L., Martik, B.N., Salman, F., Warner, L.T., Valla, S.V., Bock, D.D., and Dacks, A.M. (2020). The wiring logic of an identified serotonergic neuron that spans sensory networks. bioRxiv. https://doi.org/10.1101/2020.02.24.963660.

73. Horne, J.A., Langille, C., McLin, S., Wiederman, M., Lu, Z., Xu, C.S., Plaza, S.M., Scheffer, L.K., Hess, H.F., and Meinertzhagen, I.A. (2018). A resource for the *Drosophila* antennal lobe provided by the connectome of glomerulus VA1v. eLife *7*, e37550.

74. Patella, P., and Wilson, R.I. (2018). Functional Maps of Mechanosensory Features in the Drosophila Brain. Curr. Biol. *28*, 1189–1203.e5.

75. Pacheco, D.A., Thiberge, S.Y., Pnevmatikakis, E., and Murthy, M. (2019). Auditory Activity is Diverse and Widespread Throughout the Central Brain of Drosophila. bioRxiv. https://doi.org/10.1101/709519.

76. Suver, M.P., Matheson, A.M.M., Sarkar, S., Damiata, M., Schoppik, D., and Nagel, K.I. (2019). Encoding of Wind Direction by Central Neurons in Drosophila. Neuron *102*, 828–842.e7.

77. Robie, A.A., Hirokawa, J., Edwards, A.W., Umayam, L.A., Lee, A., Phillips, M.L., Card, G.M., Korff, W., Rubin, G.M., Simpson, J.H., et al. (2017). Mapping the Neural Substrates of Behavior. Cell *170*, 393–406.e28.

78. Galizia, C.G., Münch, D., Strauch, M., Nissler, A., and Ma, S. (2010). Integrating heterogeneous odor response data into a common response model: A DoOR to the complete olfactome. Chem. Senses *35*, 551–563.

79. Zhao, B., Sun, J., Zhang, X., Mo, H., Niu, Y., Li, Q., Wang, L., and Zhong, Y. (2019). Long-term memory is formed immediately without the need for protein synthesis-dependent consolidation in Drosophila. Nat. Commun. *10*, 4550.

80. Sayin, S., De Backer, J.-F., Siju, K.P., Wosniack, M.E., Lewis, L.P., Frisch, L.-M., Gansen, B., Schlegel, P., Edmondson-Stait, A., Sharifi, N., et al. (2019). A Neural Circuit Arbitrates between Persistence and Withdrawal in Hungry Drosophila. Neuron *104*, 544–558.e6.

81. Frechter, S., Bates, A.S., Tootoonian, S., Dolan, M.-J., Manton, J.D., Jamasb, A., Kohl, J., Bock, D., and Gregory, S.X. (2019). Functional and Anatomical Specificity in a Higher Olfactory Centre. eLife. *8*, e44590.

82. Li, P.H., Lindsey, L.F., Januszewski, M., Zheng, Z., Bates, A.S., et al. (2019). Automated reconstruction of a serial-section EM Drosophila brain with flood-filling networks and local realignment. bioRxiv. https://doi.org/10.1101/605634.

83. Saalfeld, S., Cardona, A., Hartenstein, V., and Tomancak, P. (2009). CATMAID: collaborative annotation toolkit for massive amounts of image data. Bioinformatics *25*, 1984–1986.

84. Schneider-Mizell, C.M., Gerhard, S., Longair, M., Kazimiers, T., Li, F., Zwart, M.F., Champion, A., Midgley, F.M., Fetter, R.D., Saalfeld, S., and Cardona, A. (2016). Quantitative neuroanatomy for connectomics in Drosophila. eLife *5*, e12059.

85. Ohyama, T., Schneider-Mizell, C.M., Fetter, R.D., Aleman, J.V., Franconville, R., Rivera-Alba, M., Mensh, B.D., Branson, K.M., Simpson, J.H., Truman, J.W., et al. (2015). A multilevel multimodal circuit enhances action selection in Drosophila. Nature *520*, 633–639.

86. Schlegel, P., Texada, M.J., Miroschnikow, A., Schoofs, A., Hückesfeld, S., Peters, M., Schneider-Mizell, C.M., Lacin, H., Li, F., Fetter, R.D., et al. (2016). Synaptic transmission parallels neuromodulation in a central food-intake circuit. eLife *5*, e16799.

87. Prokop, A., and Meinertzhagen, I.A. (2006). Development and structure of synaptic contacts in Drosophila. Semin. Cell Dev. Biol. *17*, 20–30.

88. Busch, S., Selcho, M., Ito, K., and Tanimoto, H. (2009). A map of octopaminergic neurons in the Drosophila brain. J. Comp. Neurol. *513*, 643–667.

89. Coates, K.E., Majot, A.T., Zhang, X., Michael, C.T., Spitzer, S.L., Gaudry, Q., and Dacks, A.M. (2017). Identified Serotonergic Modulatory Neurons Have Heterogeneous Synaptic Connectivity within the Olfactory System of *Drosophila*. J. Neurosci. *37*, 7318–7331.

90. Okada, R., Awasaki, T., and Ito, K. (2009). Gamma-aminobutyric acid (GABA)-mediated neural connections in the Drosophila antennal lobe. J. Comp. Neurol. *514*, 74–91.

91. Wilson, R.I., and Laurent, G. (2005). Role of GABAergic inhibition in shaping odor-evoked spatiotemporal patterns in the Drosophila antennal lobe. J. Neurosci. *25*, 9069–9079.

92. Liang, L., Li, Y., Potter, C.J., Yizhar, O., Deisseroth, K., Tsien, R.W., and Luo, L. (2013). GABAergic projection neurons route selective olfactory inputs to specific higher-order neurons. Neuron *79*, 917–931.

93. Lai, S.-L., Awasaki, T., Ito, K., and Lee, T. (2008). Clonal analysis of Drosophila antennal lobe neurons: diverse neuronal architectures in the lateral neuroblast lineage. Development *135*, 2883–2893.

94. Kohl, J., Ostrovsky, A.D., Frechter, S., and Jefferis, G.S.X.E. (2013). A bidirectional circuit switch reroutes pheromone signals in male and female brains. Cell *155*, 1610–1623.

95. Hartenstein, V., Omoto, J.J., Ngo, K.T., Wong, D., Kuert, P.A., Reichert, H., Lovick, J.K., and Younossi-Hartenstein, A. (2018). Structure and development of the subesophageal zone of the Drosophila brain. I. Segmental architecture, compartmentalization, and lineage anatomy. J. Comp. Neurol. *526*, 6–32.

96. Urbach, R., Jussen, D., and Technau, G.M. (2016). Gene expression profiles uncover individual identities of gnathal neuroblasts and serial homologies in the embryonic CNS of Drosophila. Development *143*, 1290–1301.

97. Bräcker, L.B., Siju, K.P., Varela, N., Aso, Y., Zhang, M., Hein, I., Vasconcelos, M.L., and Grunwald Kadow, I.C. (2013). Essential role of the mushroom body in context-dependent $CO_2$ avoidance in Drosophila. Curr. Biol. *23*, 1228–1234.

98. Stepanyants, A., Tamás, G., and Chklovskii, D.B. (2004). Class-specific features of neuronal wiring. Neuron *43*, 251–259.

99. Lafarge, T., Pateiro-López, B., Possolo, A., and Dunkers, J. (2014). R Implementation of a Polyhedral Approximation to a 3D Set of Points Using the $\alpha$-Shape. (American Statistical Association).

100. Schlager, S. (2017). Statistical Shape and Deformation Analysis (Elsevier), pp. 217–256.

101. Laissue, P.P., Reiter, C., Hiesinger, P.R., Halter, S., Fischbach, K.F., and Stocker, R.F. (1999). Three-dimensional reconstruction of the antennal lobe in Drosophila melanogaster. J. Comp. Neurol. *405*, 543–552.

102. Endo, K., Aoki, T., Yoda, Y., Kimura, K., and Hama, C. (2007). Notch signal organizes the Drosophila olfactory circuitry by diversifying the sensory neuronal lineages. Nat. Neurosci. *10*, 153–160.

103. Virtanen, P., Gommers, R., Oliphant, T.E., Haberland, M., Reddy, T., Cournapeau, D., Burovski, E., Peterson, P., Weckesser, W., Bright, J., et al.; SciPy 1.0 Contributors (2020). SciPy 1.0: fundamental algorithms for scientific computing in Python. Nat. Methods *17*, 261–272.

104. Olsen, S.R., and Wilson, R.I. (2008). Lateral presynaptic inhibition mediates gain control in an olfactory circuit. Nature *452*, 956–960.

# STAR★METHODS

## KEY RESOURCES TABLE

| REAGENT or RESOURCE | SOURCE | IDENTIFIER |
|---|---|---|
| Deposited Data | | |
| The Full Adult Fly Brain ssTEM dataset | [21] | https://temca2data.org/ |
| FAFB manual neuronal reconstructions | This paper [5, 14, 15, 17, 21] | https://fafb.catmaid.virtualflybrain.org/ |
| Lateral horn related neuron data including EM reconstructions, synapses and light-level neurons | This paper [14, 81] | https://github.com/jefferislab/lhns |
| Partial auto-segmentation of FAFB | [82] | FAFB-FFN1, not yet public, citation is a preprint. |
| Software and Algorithms | | |
| CATMAID: source code | [83, 84] | https://github.com/catmaid/CATMAID |
| CATMAID: user documentation | [83, 84] | https://catmaid.readthedocs.io/en/stable/user.html |
| CATMAID: administrator documentation | [83, 84] | https://catmaid.readthedocs.io/en/stable/administrator.html |
| CATMAID: developer documentation | [83, 84] | https://catmaid.readthedocs.io/en/stable/developer.html |
| NBLAST | [85] | https://github.com/jefferislab/nat.nblast |
| CATMAID-to-Blender | [86] | https://github.com/schlegelp/CATMAID-to-Blender |
| natverse, including the *rcatmaid* package to interact programmatically with CATMAID | [37] | http://natverse.org/ |
| NAVis, Python neuron analysis and visualization library | [37] | https://github.com/schlegelp/navis |
| Pymaid, Python library for interacting with CATMAID | This paper | https://github.com/schlegelp/pymaid |
| FAFBseg, Python code for working with the partial auto-segmentation of FAFB from 86 | This paper | https://github.com/flyconnectome/fafbseg-py |

## RESOURCE AVAILABILITY

### Lead Contact

Further information and requests for resources should be directed to and will be fulfilled by the Lead Contact, G.S.X.E. Jefferis (jefferis@mrc-lmb.cam.ac.uk).

### Materials Availability

This study did not generate new unique reagents.

### Data and Code Availability

Neuronal reconstructions presented in this study are available in a public CATMAID instance hosted by Virtual Fly Brain (https://fafb.catmaid.virtualflybrain.org/). Neuron skeletons, synapse and metadata are provided as supplemental files. We have also made this data available in our R package *lhns* (https://github.com/jefferislab/lhns) for easy use with programmatic tools, such as the *natverse* (http://natverse.org/) [37]. Morphological and connectivity analyses were performed in R (the natverse, http://natverse.org/) and in Python (pymaid, https://github.com/schlegelp/pymaid) using open source packages maintained by the authors [37]. Code used for specific analyses will be made available from the corresponding author on request.

## METHOD DETAILS

### Neuronal Reconstruction

Reconstructions are based on a ssTEM (serial section transmission electron microscope) dataset comprising an entire female adult fly brain (FAFB, https://fafb.catmaid.virtualflybrain.org/, https://temca2data.org) (x,y,z resolution 4 nm x 4nm x 40 nm). Generation of this dataset was described previously by Zheng et al. [21]. Neurons were manually reconstructed using a modified version of CATMAID (http://www.catmaid.org) [83, 84], a Web-based environment for working on large image datasets that has been optimized for tracing and online analysis of neuronal skeletons [84]. Synapses annotated represent fast, chemical synapses matching

previously described criteria: thick, dark active zone, presynaptic (T-bars, vesicles) membrane specializations and a synaptic cleft [87]. We scored each continuous synaptic cleft as a single presynapse regardless of its size or the number of associated T-bars. Adjacent neuronal membranes in contact with the synaptic cleft were defined as being postsynaptic to a given presynaptic site. PN dendrites were reconstructed to 'identification' whereas left-hand-side axons were reconstructed to 'completion'. A subset of lateral horn neurons and WEDPNs were reconstructed to completion. MBONs, centrifugal neurons and neurons identified from tracing upstream of completed cells, were reconstructed to identification.

In general, reconstruction to 'identification' meant tracing (at least) a neuron's microtubule-containing backbone in search of major landmarks: in case of PN candidates, for example, we sought to find (a) the soma, (b) axonal projections and (c) dendrites in the antennal lobe in the first pass, such that the neuron's hemilineage and neuropil-neuropil projections could be discerned. Reconstruction to 'completion' followed the tracing protocol established by Schneider-Mizell et al. [84]. In brief, their iterative reconstruction method consists of an initial reconstruction of the entire arbour including annotation of chemical synapses, followed by edits/proofreading by the same or a different tracer. This approach was shown to produce almost no false-positives and to be effective at minimizing false-negatives, and has been used by various studies [21, 84, 85]. We modified this protocol for focused proofreading using a new partial automatic segmentation of the dataset [82]. To help us proofread extant neurons, we semi-automatically looked for missing arbour by using our manual tracings to concatenate disparate, automatically reconstructed neuron meshes. For this purpose, we wrote custom tools in both R and Python (https://github.com/jefferis/fafbseg; https://github.com/flyconnectome/fafbseg-py). Our combined effort to reconstruct the olfactory PNs cost 2180 h of reconstruction time, 690 h of arbour review time and created 54 cm of cable. We estimate that is only ~4% of the LH's neural cable.

### Choosing Neurons to Reconstruct

Broadly, there were two approaches when looking for specific individual neurons/cell types: (a) By identifying anatomical loci in the EM that corresponded to anatomical features for our identified lateral horn neurons, for example, their cell body fibers. We bridged between loci identified at light-level and the EM in order to build a list of candidate neurons. (b) We used NBLAST to search for our MCFO derived morphologies against extant LH tracing in this dataset. Over the past ~2 years a community of researchers across the world have been reconstructing neurons in FAFB, allowing us to, with their consent, NBLAST against thousands of partial reconstructions to build upon our candidate list for each cell type (see Acknowledgments). Once traced, neurons could be matched to describe light-level data, and so more accurately identified. We have also attempted to identify the secondary, larval-born lineage of origin for most of our reconstructions, where they were not suspected to be embryo-born primary neurons. There are two naming conventions for a set of about the same ~100 lineage clones, that have originated in the groups of K. Ito and T. Lee, and V. Hartenstein respectively [25–28]. We give both names in our Data S3 and S4, and use the 'Hartenstein' names in this text.

### Antennal Lobe Projection Neurons (AL PNs)

We generated a full catalog of adult AL PNs by reconstructing all neurons in the three major (mALT, mlALT and lALT) and the heterogeneous transversal (tALT) antennal lobe tracts on the fly's right brain hemisphere [21–23, 81]. For this, we chose multiple cross sections through the base of each tract and reconstructed all neurons within it to identification. For completeness, we note the serotonergic CSD neuron which we excluded from our list because its unpolarised nature precludes it from being classified as a classical projection neuron [72, 88, 89].

### Lateral Horn Neurons (LHNs)

The large number of ~1400 lateral horn neurons (LHNs), meant it was not feasible to reconstruct the entire set even to identification. We chose to reconstruct a sample of 82 to completion. 26 of these reconstructions have previously been reported [15, 17]. These and a further 11 reconstructions correspond to neurons that can be experimentally targeted by specific genetic drivers [14]. 19 further LHNs were semi-randomly chosen because they represent a range of different morphologies. We matched them to 42 defined cell types [8, 57] (Figure S4A) and found six new cell types (Data S1 and S4).

### LH Centrifugal Neurons

These neurons were discovered as a by-product of our upstream tracing work. In order to make sure we had as many as possible, we also searched the cell body fibers from which these 10 neurons derive, and the heterogeneous tract connecting the LH and MB. We could not identify additional LH centrifugal neurons, though more may exist that do not take these tracts or connect onto PN axons. We cannot be sure that we have found all members of this class in this sparsely reconstructed dataset.

### Wedge Projection Neurons (WEDPNs)

For the purpose of this study, WEDPNs are defined as neurons with neurites in both the Wedge and/or its surrounding neuropils, and the LH. To find more WEDPNs than we had previously described [14], we sought to target the tract they take into the LH in FAFB (the trans-lALT, ~100 candidates) and the four hemilineages from which they derive (~200 candidates). By tracing these candidates to identification, we identified 24 WEDPNs. All belonged to GABAergic lineages [25]. A further ~10 neurons from the wedge brushed past the LH, including previously studied wind-sensitive WEDPNs [76]. We found that all types except type 7 had an axon in the LH and dendrites in the wedge, with spill-over into other ill-defined third-order mechanosensory neuropils in the inferior region of the ventrolateral protocerebrum (Figure 6D). Neurons of cell type WEDPN7 could be considered LHONs, but we do not treat them as such for analyses in this study; they differ from our core LHON set in that they do not get uPN input though they receive input in the LH from cholinergic mPNs and LHNs (data not shown).

### Kenyon Cells

We examined 15 Kenyon cells, whose axons had previously been constructed to identification and dendrites to completion [21]. We reviewed these constructions to make them synapse-complete.

### *Mushroom Body Output Neurons (MBONs)*

To identify MBON inputs to LH centrifugal neurons, we transformed segmented neurons from a light-level study into FAFB space [10], and focused on certain MBONs that appeared to overlap with our candidate centrifugal neuron reconstructions (data not shown). After locating the mushroom body compartment and these MBONs' dendrites, we then targeted their axons for synaptic reconstruction in the vicinity of centrifugal neurons.

### *Upstream Tracing from defined Neurons*

In several cases, we sought to characterize the neurons upstream of cells of interest. We needed to reconstruct upstream neurons to identification in order to discover their class [57], where classes included ORNs, uPNs, mPNs, AL local neurons, LHONs, LHLNs, MBONs, KCs, WEDPNs, AMMC local neurons, AMMC projection neurons, LHPV12a1, LH centrifugal neurons and others. For the reconstruction upstream of 6 fully reconstructed LHNs (Figure S5A), we attempted to reconstruct all synaptic inputs, successfully connecting 93% to an upstream neuron that could be classed. Our LHN sample was necessarily small due to the time consuming nature of this work, ~80 h per neuron. To find neurons upstream of mPN dendrites, we sampled fully from one GABAergic mPN dendrite (600 inputs) and a random 25% from a larger, cholinergic mPN dendrite (500/2000 inputs). We chose random sampling because we were interested in the distribution of synaptic partners across glomeruli. In case of reconstructing upstream of all olfactory PN axons in the LH, and WEDPN arbours, we adopted a more efficient sampling strategy to find strongly connected partners, which can be biased by the class identity of that partner [82]. Briefly, this strategy uses a recent partial auto segmentation of the volume to rank potentially connected segments by the numbers of connections they have with the starter neuron, and a human tracer goes through the ranked list. We reconstructed from all such segments predicted to connect by 2 or more synapses with our starter neurons, which focused us away from weak single-synapse unitary connections. We covered 79% of synaptic inputs to PN axons, and 69% of our chosen 4 WEDPNs. Upstream neurons were sometimes assigned a putative modality (e.g., gustatory) if they appear to have dendrites in brain regions known to process particular sensory information (e.g., the subesophageal zone).

## Bridging EM and light-level Data

In order to assign glomerular identity to the PNs, we used NBLAST [49] to compare the FAFB EM PNs with segmentations of annotated PNs in the light-level FlyCircuit database ([30, 49]; www.flycircuit.tw). We used a linear, followed by a non-rigid transformation to bring neurons from FAFB into FCWB (fly circuit whole brain) space [49]. Having EM and light-level neurons in the same space, we performed an all-by-all NBLAST and looked for the closest match in the FlyCircuit database. For the majority of FAFB PNs we were able to find an intuitive match. Identity of PNs types not in the FlyCircuit database (e.g., DP1m) and in cases of ambiguous NBLAST results were manually verified by comparing PN morphology (antennal lobe dendrites, LH arbours and lineage) to published data [57].

## PN Types and Putative Neurotransmitters

PNs and the lineages to which they belong have been extensively studied in the literature. This allowed us to cross-reference most reconstructed PNs with extant data. In cases of previously unknown PNs, we gave them new type names conforming to the widely used "trivial" names adPN, lPN and vPN (see Figure S1A, Data S3 and Video S2). In absence of any developmental data, these new trivial names are based entirely on soma position and cell body fiber and do not correlate with lineages. To assign putative neurotransmitters, we assumed that neurons within the same hemilineage express the same neurotransmitter(s). This has been shown to be true in the ventral nerve cord [24]. Most extant transmitter data is based on GAL4 driver lines which can have incomplete (i.e., only a subset of a given PN type) or overlapping (i.e., multiple PN types) expression patterns. In addition, individual studies often test only single neurotransmitters and do not show negative staining. We collated this available data and assigned putative neurotransmitters. In general, neurons contained in the mALT and lALT were shown to be cholinergic based on immuno-histochemical stainings [23]. At the same time, "few if any iACT [mALT] and oACT [lALT] PNs express Gad1" [90]. By itself we did not consider this sufficient evidence for assigning transmitters. Instead we sought additional immuno-histochemical or physiological data:

### *adPN (lineage BAMv3)*

PNs in this anterodorsal lineage project through the mALT suggesting they might be cholinergic. In addition, they do not show GABA immuno-reactivity [91]. In electrophysiological experiments neurons in this lineage excite downstream neurons in the lateral horn [18, 20]. We therefore assigned Acetylcholine as putative neurotransmitter.

### *vPN (lineage BAla1)*

This ventral lineage contains all PNs of the mlALT and was previously shown to be GABAergic based on immuno-histochemical stainings [92]. Additionally, the mlALT shows no immuno-reactivity for ChAT [22]. Based on this, we assigned GABA as putative neurotransmitter.

### *lPN and l2PN (hemilineages BAlc ventral + dorsal)*

The more dorsal hemilineage of this lateral PN cluster has historically been referred to as lPNs. We refer to the more ventral hemilineage which has a clearly separate cell body fiber as l2PNs. This lineage as a whole contains both PNs and AL local neurons. At least some (likely local) neurons are GABAergic [25, 91, 93]. We also know that the lPNs in the dorsal lineage hemilineage (contained in GH146-GAL4 expression pattern) are GABA-negative [91]. In addition, the dorsal hemilineage contains uPNs that have been shown to excite downstream neurons in the lateral horn [18, 20, 94]. We, therefore, assigned Acetylcholine as putative neurotransmitter to lPNs. Because of the uncertainty with regards to l2PNs in the ventral hemilinage, we did not assign a neurotransmitter to it.

#### lvPN (lineage BAlp4/ALlv1)

PNs in this lateroventral lineage project through the mALT and trans-mALTs. It was shown to be neither GABA-, seroton-, dopamin- or octopaminergic [25]. No olfactory PN has been described as glutamatergic and a recent staining based on a sparse split-GAL4 line labeling ~12 cells in the lineage, showed them to be ChAT-positive (I. Taisz, personal communication). We therefore assigned Acetylcholine as the putative neurotransmitter for neurons in this hemilineage.

#### lv2PN

This group contains two PNs that to our knowledge have not been previously described. Their soma position is very close to that of lvPNs which is why they were called lv2PNs.

#### VUMa2 PNs

These ventral unpaired medial neurons were shown to be octopaminergic [88]. We note the occurrence of both small clear core and large dense-core vesicles in these neurons, possibly indicative of a second neurotransmitter [86].

#### Inferior PNs: ilPN, il2PN, ivPN, imPN

These PNs originate in the subesophageal zone (SEZ) where they form several distinct clusters. A recent survey of secondary lineages in this region did not identify any that give rise to olfactory PNs [95]. Only a minority of subesophageal lineages divide in the larva to form new adult neurons (28/180), and half have stopped dividing by the late embryonic stage [96]. Therefore, it appears that many small lineages of primary neurons are made in the subesophageal zone, and they likely include some of our PNs, including the VUM cluster. Indeed, similar neurons exist in the larva [59]. We are currently unable to identify exactly which neuroblasts made these PNs. Hence, we assigned new trivial names and included an "i" prefix for "inferior" to indicate their origin in the SEZ. The bilateral V PNs in the ilPN cluster bilaterally innervate the V glomerulus and were shown to be cholinergic but not GABA-, dopamin- or serotonergic [97]. For other PNs in these clusters we did not find transmitter data.

#### Superior PNs: sdPN, spPN

Although these neurons might well be secondary neurons, we are currently unable to associate them with a lineage. No transmitter was assigned.

### Connectivity Modeling

We explore three different models with increasing complexity for predicting connectivity. The first two methods use only neuron morphology, 'overlap score' and assessment of proximal 'potential synaptic contact sites'. The third method, 'presynapses within range', uses proximity to presynapses (outputs).

#### Overlap Score

In order to quantify the overlap between neuronal skeletons for PNs and LHNs, derived from both light-level and EM data we employed the following 'overlap' score [8]:

$$f(i_s, j_k) = \sum_{k=1}^{n} e^{-d^2/2\delta^2}$$

Skeletons were resampled so that we considered 'points' in the neuron at 1 $\mu$m intervals and an 'overlap score' calculated as the sum of $f(i_{s,\,jk})$ over all points $s$ of $i$. Here, $i$ is the axonal portion of a neuron, $j$ is the dendritic portion of a putative target, $\delta$ is the distance between two points at which a synapse might occur (e.g., 1 $\mu$m), and $d$ is the euclidean distance between points s and k. The sum was taken of the scores between each point in i and each point in j. The value for delta ($\delta$) was determined empirically by using our pool of projection neurons and their downstream neurons and taking the 90% quantile for distance between observed connections, i.e., the distance between a given presynapses and the downstream neuron's skeleton nodes (generally placed on the neuron's center line): 1210 nm. Light-level reconstructions used in Figure 4E stem from stochastic labeling experiments [14, 30], that have been previously been registered from hundreds of brains to a common template, categorised and identified [8, 49].

#### Potential Synaptic Contact Sites

This model used a previously published algorithm for detecting potential synapses [98]. In brief, this approach identifies sites of potential contact as locations where an axonal branch of a source neuron is present within a certain distance of a dendritic branch of a target neuron. We again used the value of 1210 nm.

#### Presynapses within Range (near synapses)

In contrast to the two prior methods, this method takes identified presynapses (outputs) on the source neuron into account and asks whether a target neuron is close enough to a given presynapse for this to be a potential synaptic contact between both. To assess 'proximity' to a presynapse, we used the same delta value as for the overlap score model, 1210 nm. When predicting connections, any presynapse from the potential upstream pool (e.g., all olfactory PNs) within this distance to a target neuron, is predicted to connect.

### Compartmentalising Neurons

Fully reconstructed neurons were segregated into axon and dendrites using a centrifugal synapse flow centrality algorithm [84], counting polyadic presynapses (outputs) once. We verified that neurons were suitably polarized by calculating their axon-dendrite segregation index [84] which is a quantification for the degree of segregation of postsynapses and presynapses (0, totally unsegregated; 1, completely polarized). The mean ± SD segregation index for LHPD2a1/b1 neurons was 0.27 ± 0.09 indicating that these

neurons are polarized but receive heavy axo-axonic modulation as well as outputting significantly in the lateral horn. MBONs are highly polarized, for example the right-side MBON-$\alpha$2sc has a segregation index of 0.72.

## Morphological Analysis

Morphological analysis for this paper was done using the natverse (R, https://github.com/natverse) [37] and pymaid and its associated libraries (Python, https://github.com/schlegelp/pymaid). Morphological clustering using NBLAST [49] was performed on either the dendritic and/or the axonal arbours of neuronal skeletons. Cell body fibers and the primary dendrites connecting dendritic and axonal arbours were removed because their fasciculation, especially in the single EM brain space, made NBLAST less sensitive to dendritic and axonal differences. Clustering was performed using functions for hierarchical clustering in base R on euclidean distance matrices of NBLAST scores, employing Ward's clustering criterion. In order to determine the volumes and surface areas of neurons, we stitched together fragmented neuron volumes from a recent auto-segmentation of the FAFB dataset [82]. Cohesive volumes were made using the R package *alphashape3d* [99]. Metrics were calculated using the R package, *Rvcg* [100].

## Antennal Lobe Model

The antennal lobe (AL) consists of 51 olfactory and 7 non-olfactory glomeruli [5]. There has been some confusion about glomerulus VM6 and VC5 in the field and indeed it is very likely that different studies have given the same neurons different names and different neurons the same name. We believe that what we have listed as VC5 has been previously referred to as VM6 [101, 102], or as VM6+VP1 [29, 31] or as "VC5 and VM6" [23]. We find that VC5 is a large glomerulus with three uPNs from two different lineages. From the soma position and axon morphology, we think one of these uPNs is an embryonic anterodorsal PN similar to the VM6+VP1 PN described by Yu et al. (2010) [29]. We also note that there is some indication that VC5 might not be olfactory but rather thermo- or hygrosensory [5].

Each glomerulus is defined by a specific set of sensory neurons. Because the total number of sensory neurons per AL exceeds 1000 [31], we found it impractical to reconstruct them to define glomerular boundaries. Hence, we used the dendrites of previously described, "canonical" uniglomerular PNs (uPN) whenever possible and only fall back to sensory neurons for glomeruli without known uPNs. uPN dendrites (and sensory neuron axons) can have extra-glomerular branches which complicates defining cohesive, non-overlapping glomerular compartments. In addition, we found that dendrites of multi-glomerular PNs often appear to be in between glomeruli. We therefore chose a probabilistic approach to calculate PN-by-glomerulus innervation scores: we first pruned all PNs to their dendrites and generated evenly sampled point clouds. We then used the point clouds of known uPNs (sensory neuron synapses for glomeruli VP1-5 [5]) to generate 3D Gaussian kernel density estimates (KDE) (Figure 1C). To validate the KDEs we reconstructed at least 2 sensory neurons for each glomerulus to synapse-completion. This effort yielded ~32,000 synapse positions for which we predicted a glomerulus using the PN-derived KDEs. Comparing the prediction to the ground truth, we find that 82.4% of the synapses had been assigned to the correct glomerulus and only 5.4% had been assigned to the incorrect glomerulus. The remaining 12.2% had an overall low probability and were therefore not assigned to any glomerulus (we used the same probability threshold for all subsequent analyses). See Data S2 for an AL glomerulus atlas and S1 for glomeruli meshes made using these 3D KDEs.

### *PN Classification*

We sought to broadly classify the PNs in a data-driven way. For this, we applied the KDEs' probability density functions (PDF) to the dendrite point clouds of all PNs, thereby assigning to each point probabilities for it being in a given glomerulus. To generate a PN-by-glomerulus innervation matrix, the probabilities were summed up per glomerulus and normalized. The resulting AL innervation matrix reflects the fraction of a PN's dendrites in a given glomerulus (see also Data S1). To stabilize our classification we pre-clustered PNs using NBLAST and assigned tentative "morphology types" which should approximate cell types. Next, we generated an innervation vector for each PN by sorting its innervation scores by strength: [top glomerulus, 2nd, 3rd, ..., 58th]. These vectors were averaged within each morphology type, generating a 184 by 58 matrix which was used to calculate pairwise Euclidean distances between all morphology types. Hierarchical clustering using average linkage produced two distinct superclusters that correspond to uni- and oligo-, and multiglomerular PNs (Figure 1E). The sub-clusters intuitively correspond to uni, uni+, oligo, multi and pan-glomerular PNs. Without a second dataset to validate these finer classes, we opted to group PNs into uni- (uni, uni+) and multi- (oligo, multi, pan) glomerular. Based on this, our provisional estimate of mPN cell types is ~100, however this will need to be compared across brains as it is unclear whether they, unlike at least some AL local neurons [60], are stereotyped between brains.

### *Anatomical transfer function*

To explore the contribution of individual glomeruli to computations in higher brain areas (Figures 2F and 2G and S2D, E), we fractionally attributed each PN's axonal synapses by the innervation of its dendrites: e.g., synapses of a DA1 uPN would count to 100% toward the DA1 glomerulus while the synapses of an mPN would be split across multiple glomeruli. In mathematical terms: we used the dot product of the PN-by-glomerulus innervation matrix and a vector containing the PN synapse counts.

## Network Analysis

### *Reciprocity*

We implemented a previously described metric for weighted reciprocity to measure the hierarchy of connections in the axo-axonic PN-PN network [34]. In brief, the weighted reciprocity is defined as the fraction of reciprocated edge weights $\omega$: a low reciprocity means that connections between neurons tend to be directed (or one-way) and vice versa. Consider two neurons *i* and *j* that are

connected with $\omega_{ij}^{\rightarrow} = 5$ and $\omega_{ji}^{\rightarrow} = 2$. The reciprocal portion of that connections is $\omega^{\leftrightarrow} = min(5, 2) = 2$ and the total weight is $\omega = \sum(5, 2) = 7$. With this the reciprocity is $r = \frac{\omega^{\leftrightarrow}}{\omega} = 2/7 = 0.285$. The reciprocity across the entire network is then simply the sum across all edge pairs.

#### *Local Reaching Centrality (LRC)*

The LRC of a node/neuron describes the fraction of other nodes that can be reached via outgoing connections [35]. LRC is indicative of a node's position within the hierarchy of network: a high LRC means that theoretically activity from this node can (directly or indirectly) propagate through a large part of the network; conversely, an LRC of 0 indicates that this node has no outgoing connections to other nodes in the networks and is hence a dead end. Here, we use the general implementation in the *networkx* Python package (http://networkx.github.io) for directed, weighted graphs that takes the average edge weight (i.e., synapse count) along a path into account:

$$C_R^{'}(i) = \frac{1}{N-1} \sum_{j:0 < d^{out}(i,j) < \infty} \left( \frac{\sum_{k=1}^{d^{out}(i,j)} w_i^{(k)} j}{d^{out}(i,j)} \right)$$

Here $N$ denotes the number of nodes in the graph, $d^{out}(i, j)$ is the length of the directed path that goes from $i$ to $j$ via out-going edges and $w_i^{(k)}$ is the weight of the $k$-th edge along this path.

#### *(Out-)Degree*

A node's degree (Figure S3A) is the count of all incoming and outgoing connections. The weighted out-degree (Figures S3B and S3C) of a node is the sum of the edge weights (number of synaptic connections) for edges pointing away from that node. In contrast to LRC, the out-degree only describes the immediately (i.e., directly connected) surroundings of a node. A high out-degree means that a node represents a hub that may exert strong control over its direct downstream targets.

#### *Community Detection*

Community detection in the axo-axonic PN-PN network was performed using the Python implementation of the Leiden algorithm [40] (https://leidenalg.readthedocs.io). To find a partition, we used the modularity optimization (ModularityVertexPartition) method which employs a quality function that takes edge weights and directionality into account.

## QUANTIFICATION AND STATISTICAL ANALYSIS

All statistical analyses were performed using R 3.6.0, using the package *ggpubr* (https://github.com/kassambara/ggpubr) or Python 3.7 using the *scipy* package (https://www.scipy.org) [103]. Hierarchical clustering similarity was assessed using Baker's gamma index, as implemented in the R package *dendextend* (https://github.com/talgalili/dendextend/) [47, 48]). Lifetime sparseness was calculated as in [45, 104], as implemented in the R package *gphys* (https://github.com/jefferis/gphys) or *navis* (https://github.com/schlegelp/navis) for Python. Hierarchical clustering was performed using the base R package stats or *scipy* for Python. Our tSNEs were created using the R package *Rtsne*, on centered data at a perplexity of 5 after an initial PCA step (https://github.com/jkrijthe/Rtsne). Boxplots display the 'minimum', 25th percentile, median, 75th percentile, and 'maximum' of the data shown, as is standard. We used a total of 15 Kenyon cell, 82 lateral horn neuron (26 local, 26 output), 127 cholinergic uniglomerular projection neuron, 117 cholinergic multiglomerular projection neurons, 26 GABAergic uniglomerular projection neuron and 20 GABAergic multiglomerular projection neuron FAFB reconstructions for our analyses unless otherwise specified. See Data S1 for connections and synapses.

Current Biology, Volume *30*

## Supplemental Information

# Complete Connectomic Reconstruction of Olfactory Projection Neurons in the Fly Brain

**Alexander S. Bates, Philipp Schlegel, Ruairi J.V. Roberts, Nikolas Drummond, Imaan F.M. Tamimi, Robert Turnbull, Xincheng Zhao, Elizabeth C. Marin, Patricia D. Popovici, Serene Dhawan, Arian Jamasb, Alexandre Javier, Laia Serratosa Capdevila, Feng Li, Gerald M. Rubin, Scott Waddell, Davi D. Bock, Marta Costa, and Gregory S.X.E. Jefferis**

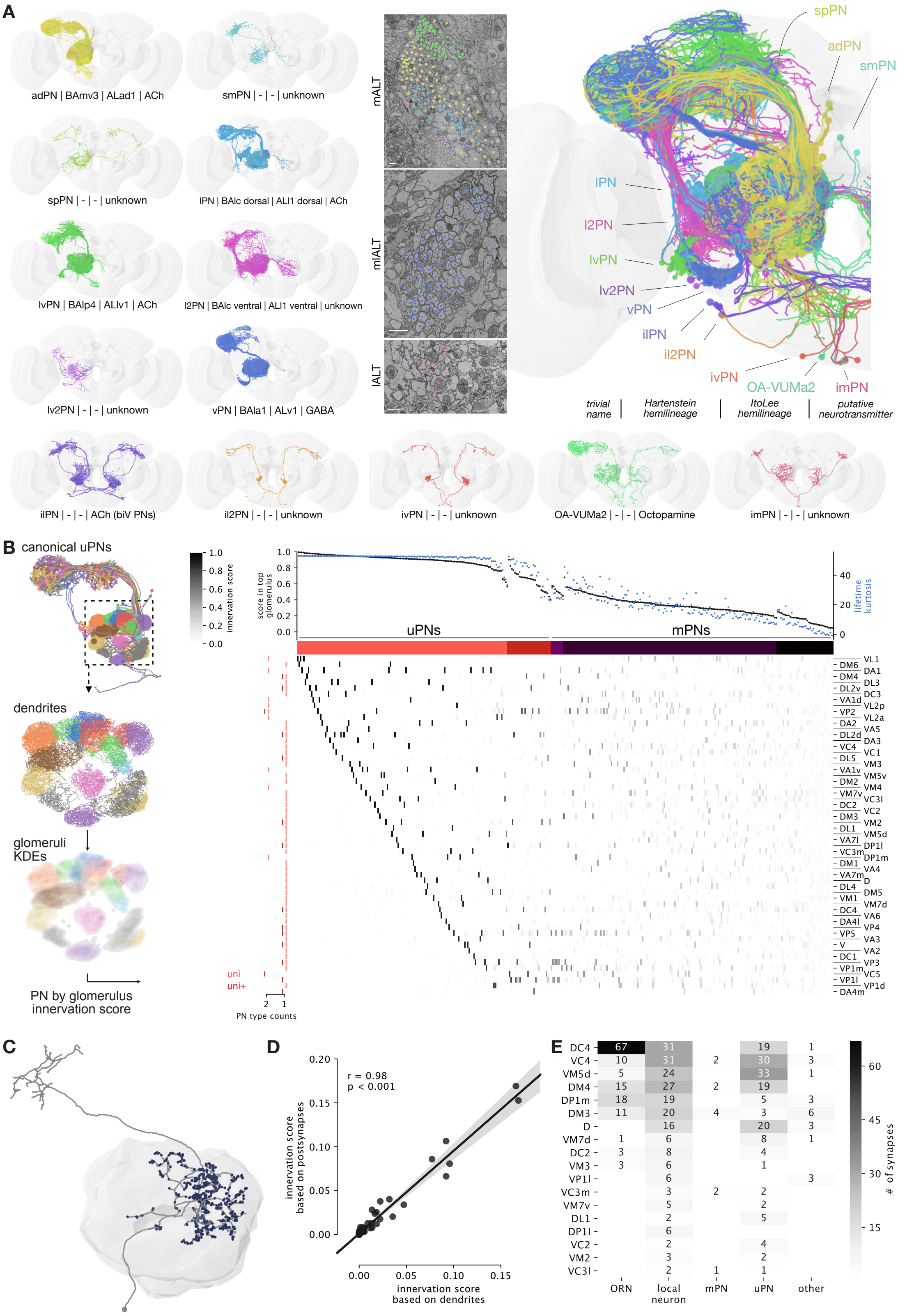


**Figure S1: PN lineages, putative transmitters and AL innervation patterns,** **Related to to Figure 1.** **A** Projection neurons (PNs) by trivial name; if available, (hemi-) lineage and putative neurotransmitters are given [S1–S4]. EM images show cross sections through the three main antennal lobe (AL) tracts (ALTs) with PN profiles highlighted (scale bar 1 micron). See also Video S1. Abbreviations: d=dorsal; i=inferior; l=lateral; m=medial; p=posterior; v=ventral. **B** PN by glomerulus innervation matrix based on probabilistic model of the AL (see Methods for details). PNs (columns) are sorted and colored according to their clustering in Figure 1E. Top scatter plot shows two metrics for PN sparseness: top innervation score (the highest value in each column) & lifetime kurtosis [S5]. Left line plot shows number of uPN (uni/uni+) types per glomerulus. **C** Single multiglomerular PN (mPN) which was traced to synapse completion in the AL. Dendritic synaptic inputs highlighted in blue. **D** Correlation of innervation score (which is based on only neural cable) and the actual synaptic inputs of the mPN in C. The strong correlation validates use of neurites as proxy for glomerular innervation (Pearson R, $p < 0.001$). **E** Synaptic inputs to dendrites of mPN in C by glomerulus and neuron type. Composition of inputs varies from glomerulus to glomerulus.

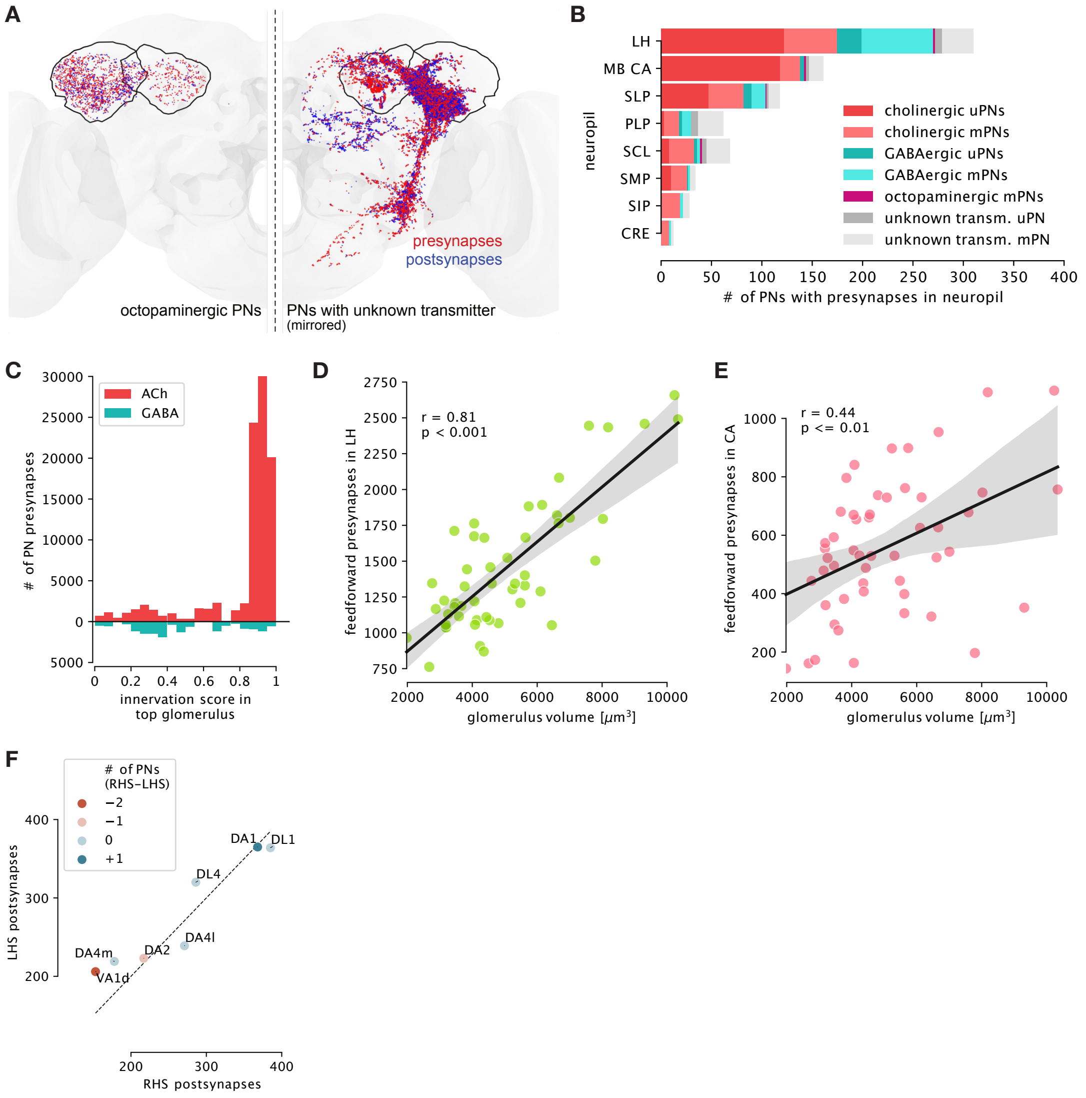


**Figure S2: Third-order targets of olfactory feedforward drive,** **Related to Figure 2.** **A** Spatial distribution of presynapses (out-puts, red) and postsynapses (inputs, blue) by octopaminergic PNs and PNs with unknown neurotransmitter. **B** Total number of PNs with >10 presynapses in target neuropil (top 8 shown). The LH is the main target of non-cholinergic and multi-glomerular PNs. **C** Choliner-gic versus GABAergic feedforward axonic presynapses by sparseness. Sparse PNs make up the majority of cholinergic presynapses while GABAergic presynapses stem from a mix of sparse and broad PNs. **D, E** Glomerulus volume versus feedforward presynapses in LH (D) and CA (E). Correlation is stronger for the LH than the CA (Pearson correlation, see STAR Methods for details). **F** Number of postsynapses between left- (LHS) and right-hand-side (RHS) homologs of 7 uPN types.

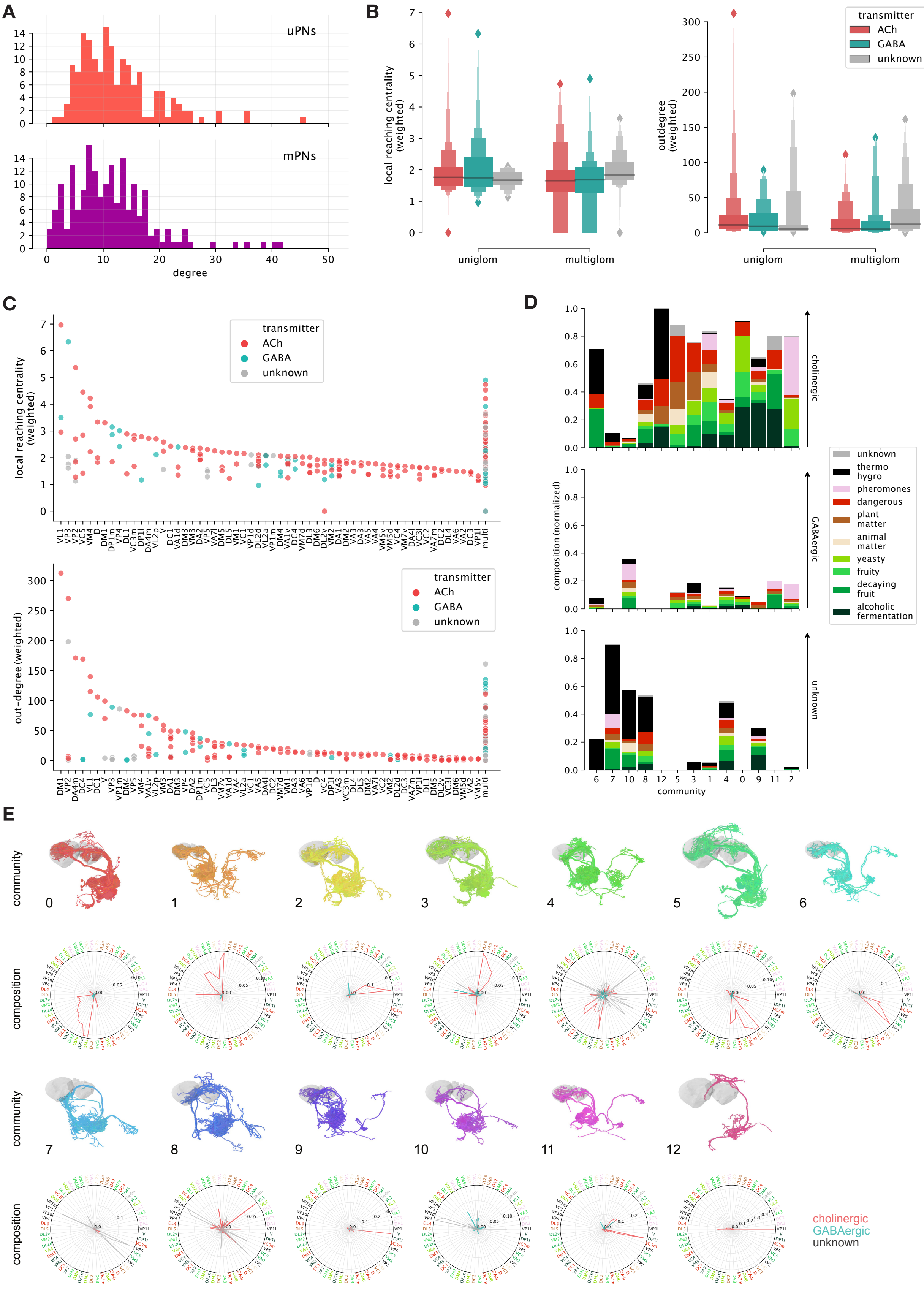


**Figure S3: Axo-axonic PN network, Related to Figure 3. A** Degree (number of all incoming and outgoing edges) distribution for uni- (top) and multiglomerular (bottom) projection neurons (PNs). **B** Local reaching centrality (LRC, top) and outdegree (bottom) does not differ between PN classes and transmitters. Individual neurons can be strong outliers though (see Figure 3F). **C** LRC (top) and out-degree (bottom) per glomerulus. Each data point represents a single PN (see also Data S3). **D** Normalised composition of axo-axonic PN-PN communities split by neurotransmitter. **E** Morphology and composition by glomerulus of individual communities. Polar plots show density per community per neurotransmitter (i.e. the area under the curves sums up to 1). For example, community 0 consists of mainly cholinergic PNs that most strongly innervate glomeruli DP1m and DM1. Colors of glomeruli labels correspond to odour scene in D.

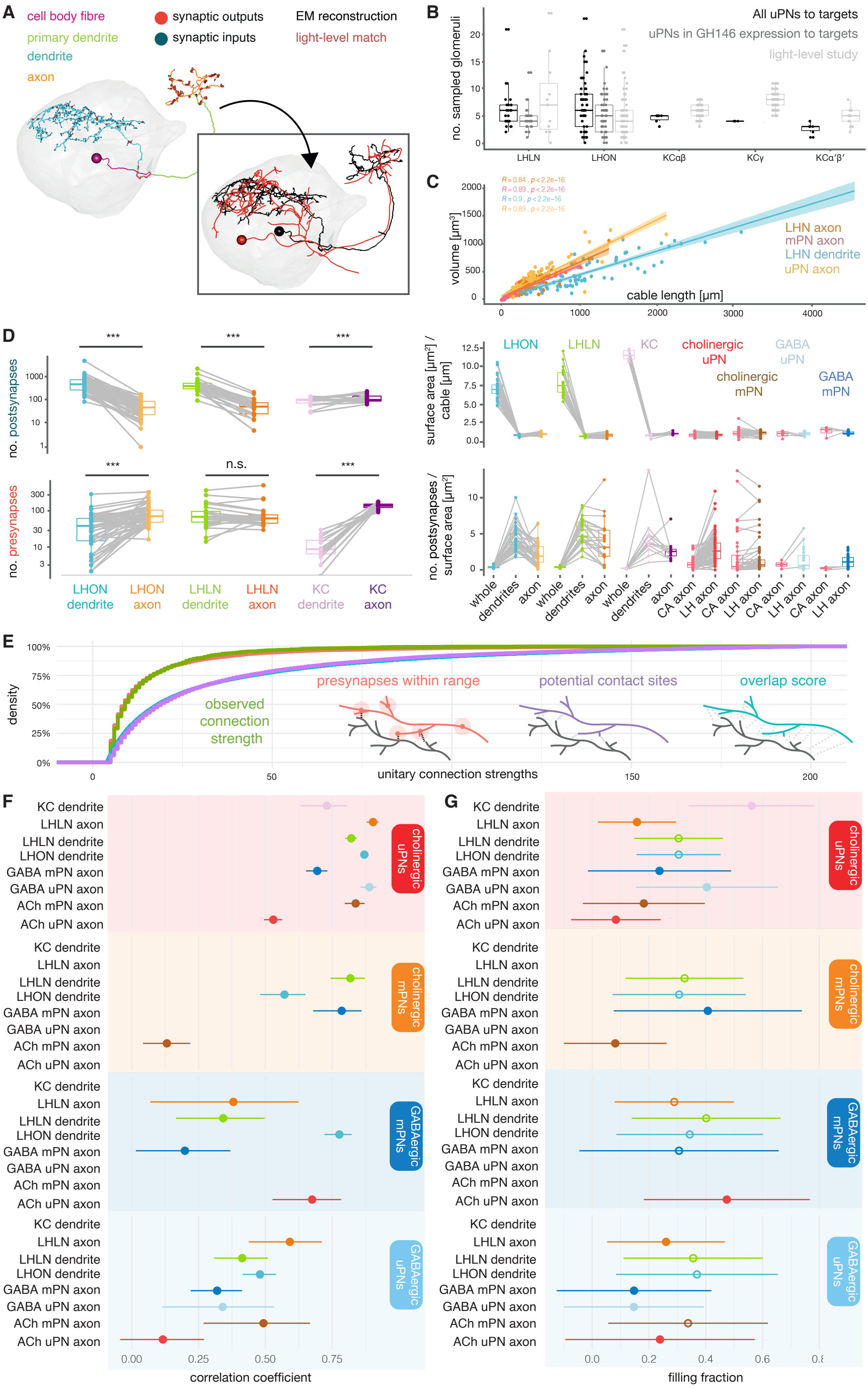
A
cell body fibre
primary dendrite
dendrite
axon
synaptic outputs
synaptic inputs
EM reconstruction
light-level match
B
All uPNs to targets
uPNs in GH146 expression to targets
light-level study
no. sampled glomeruli
LHLN
LHON
KCαβ
KCγ
KCα'β'
C
volume [μm³]
cable length [μm]
LHN axon
mPN axon
LHN dendrite
uPN axon
D
no. postsynapses
no. presynapses
LHON dendrite
LHON axon
LHLN dendrite
LHLN axon
KC dendrite
KC axon
n.s.
surface area [μm²] / cable [μm]
no. postsynapses / surface area [μm²]
LHON
LHLN
KC
cholinergic uPN
GABA uPN
cholinergic mPN
GABA mPN
whole
dendrites
axon
CA axon
LH axon
E
density
observed connection strength
presynapses within range
potential contact sites
overlap score
unitary connection strengths
F
KC dendrite
LHLN axon
LHLN dendrite
LHON dendrite
GABA mPN axon
GABA uPN axon
ACh mPN axon
ACh uPN axon
cholinergic uPNs
cholinergic mPNs
GABAergic mPNs
GABAergic uPNs
correlation coefficient
G
filling fraction

**Figure S4: Basic anatomical properties of lateral horn neurons, Related to Figure 4. A** Left, an example EM complete recon-struction of an LHON, including its synapses and separated neuron compartments [S6]. Right, this EM skeleton (black) is matched using NBLAST to a reconstructed skeleton from a library of light-level morphologies (red). This library contains defined cell types [S7], and so the EM reconstruction can be identified as AD1a1. **B** Boxplots showing the number of connected glomeruli as assessed from EM reconstruc-tions (black) and light-level studies (light grey). For LHNs, this connectivity comes from a functional assessment of uPN→LHN connections [S8], for KCs light microscopy was used [S9]. Because Jeanne *et al.* [S8] only examined PNs labeled by the driver line GH146, we also restrict our connectivity results to these neurons (dark grey). **C** Correlation between neuronal volume and cable length for dendrites and axons. **D** Right, paired boxplots comparing the numbers of pre- and postsynapses (i.e. output and input synapses) between dendrites and axons for third-order olfactory neurons' dendrites versus their axons (note log10 scale; paired Student's T-tests; *** $p <= 0.001$). Right, the surface areas of different neuronal compartments, and the number of postsynapses per unit surface area. **E** An empirical cumulative density plot displaying the unitary (i.e. single neuron to single neuron) predicted and observed connection strengths for reconstructions in our set (82 LHNs). Inset, schematic for three different ways by which synaptic connectivity may be predicted. 'Overlap score' and 'potential contact sites' do not use any synapse information, 'presynapses within range' uses only the position of output synapses. **F** The Pearson correlation coefficients for the relationship between predicted (from the 'presynapses within synaptic range' approach) and observed connections be-tween neurons. Closed circles, significant correlations ($p <= 0.05$, Student's T-test), open circles, non-significant correlations. **G** The filling fraction, the proportion of predicted synapses that are realised, is shown. Error bars, standard errors of the mean. Closed circles, significant correlations relative to LHON dendrites ($p > 0.05$, Student's T-test), open circles, non-significant correlations. Horizontal, coloured facets indicate the upstream neuron class and compartment, colours indicate downstream neuron class and compartment.

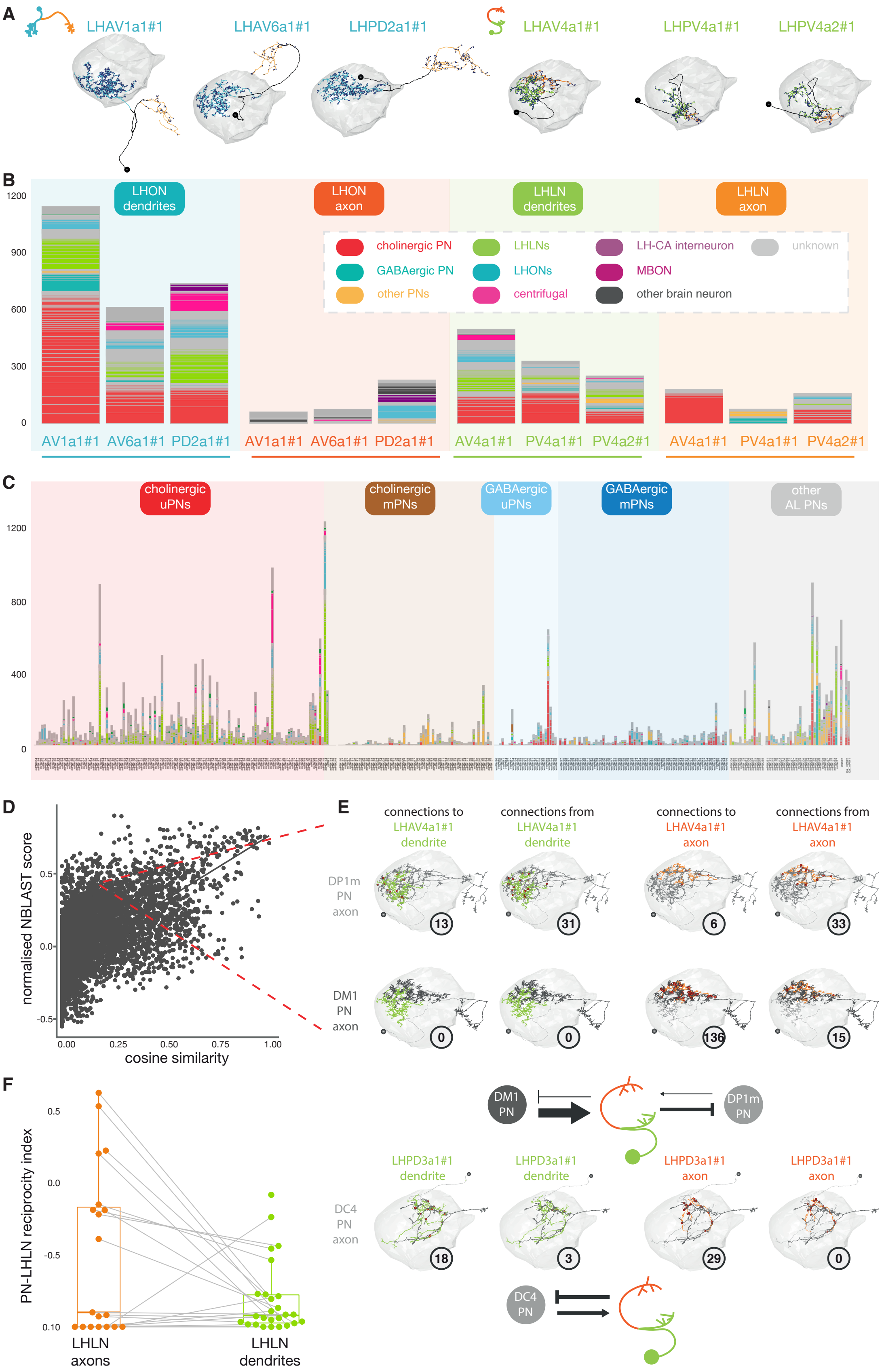


**Figure S5: The upstream connectivity of individual neurons in the lateral horn, Related to Figure 5. A** Left, the three LHONs chosen for full upstream sampling in the LH. Right, chosen LHLNs. **B** Stacked bar plots showing the result of upstream sampling in the LH for these six neurons, faceted by neuron class and compartment. **C** Stacked bar plots showing the result of upstream sampling all PNs in the LH, faceted by neuron class. **D** Scatter plot showing the correlation between PN-PN morphological similarity (NBLAST) and upstream connectivity similarity. **E** Examples of inhibitory connectivity motifs between neurons fully reconstructed in this study, with connection locations shown in red (occasionally obscure one another). Upper, an example of lateral PN-PN inhibition via an LHLN. Lower, an example of feedback inhibition. Number in circles are total connections. **F** Paired boxplot comparing the degree to which LHLN axons and dendrites reciprocate connections with PN axons. Plots show an index for connection reciprocity between LHLNs and PNs (LHLN→PN-PN→LHLN connectivity/total).

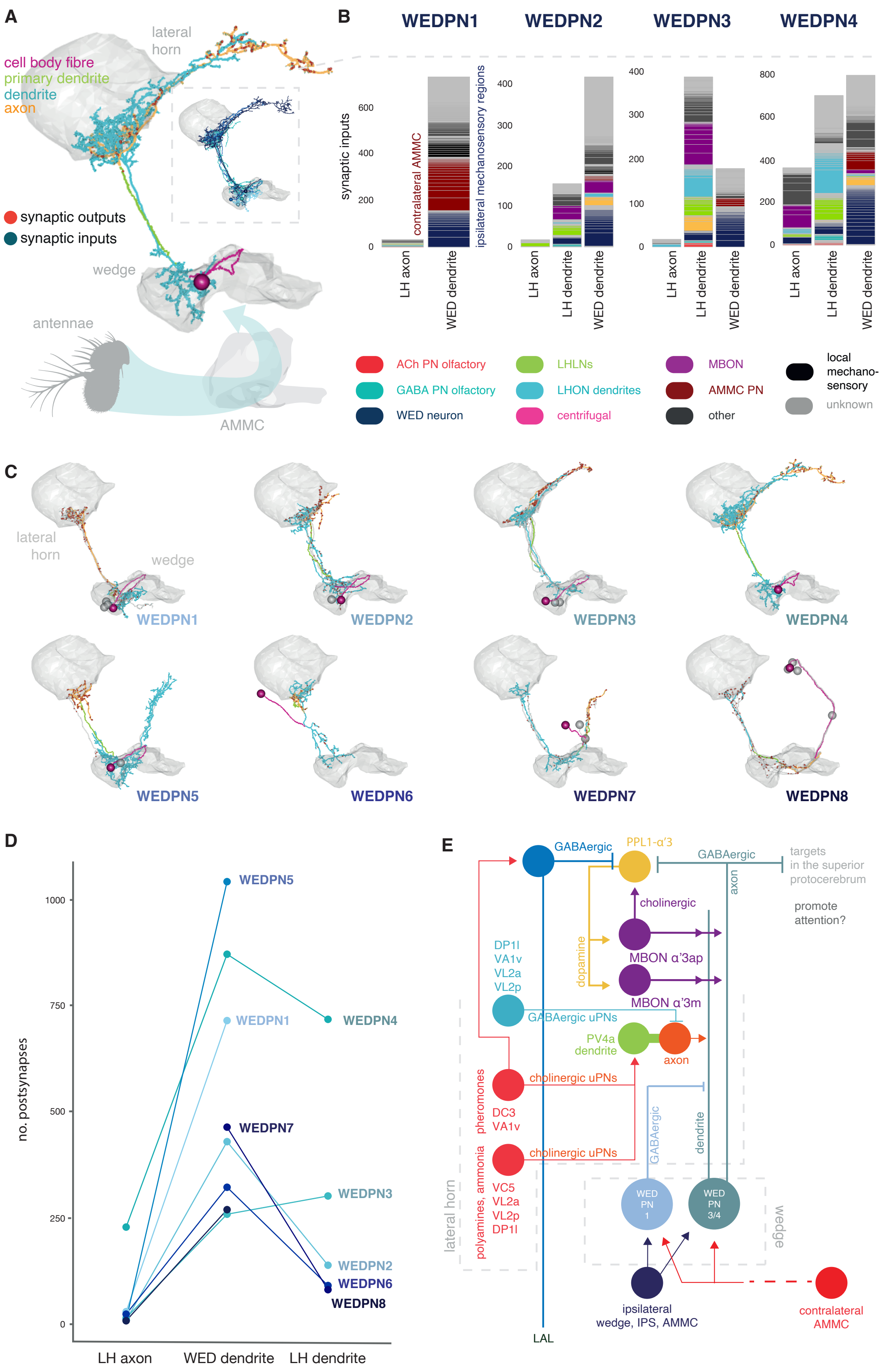

**Figure S6: Feedforward wind-sensitive and feedback memory input to the lateral horn,** Related to Figure 6. **A** Exemplary synaptic reconstruction of a WED-PN (a WED-PN4). **B** Stacked bar plots showing the result of upstream tracing from WED-PN compart-ments, for one exemplary neuron for each of four types. **C** WED-PN reconstructions built from the FAFB dataset, broken down into eight cell types. Exemplary fully reconstructed WED-PN shown in colours. **D** The number of synaptic inputs different exemplary WED-PNs, bro-ken down by neuropil and neuron compartment. Cell type colours correspond to the colours of labels in C. **E** Putative circuit integrating the results of lifetime learning, via an MBON, with specific, hardwired olfactory and mechanosensory circuitry.

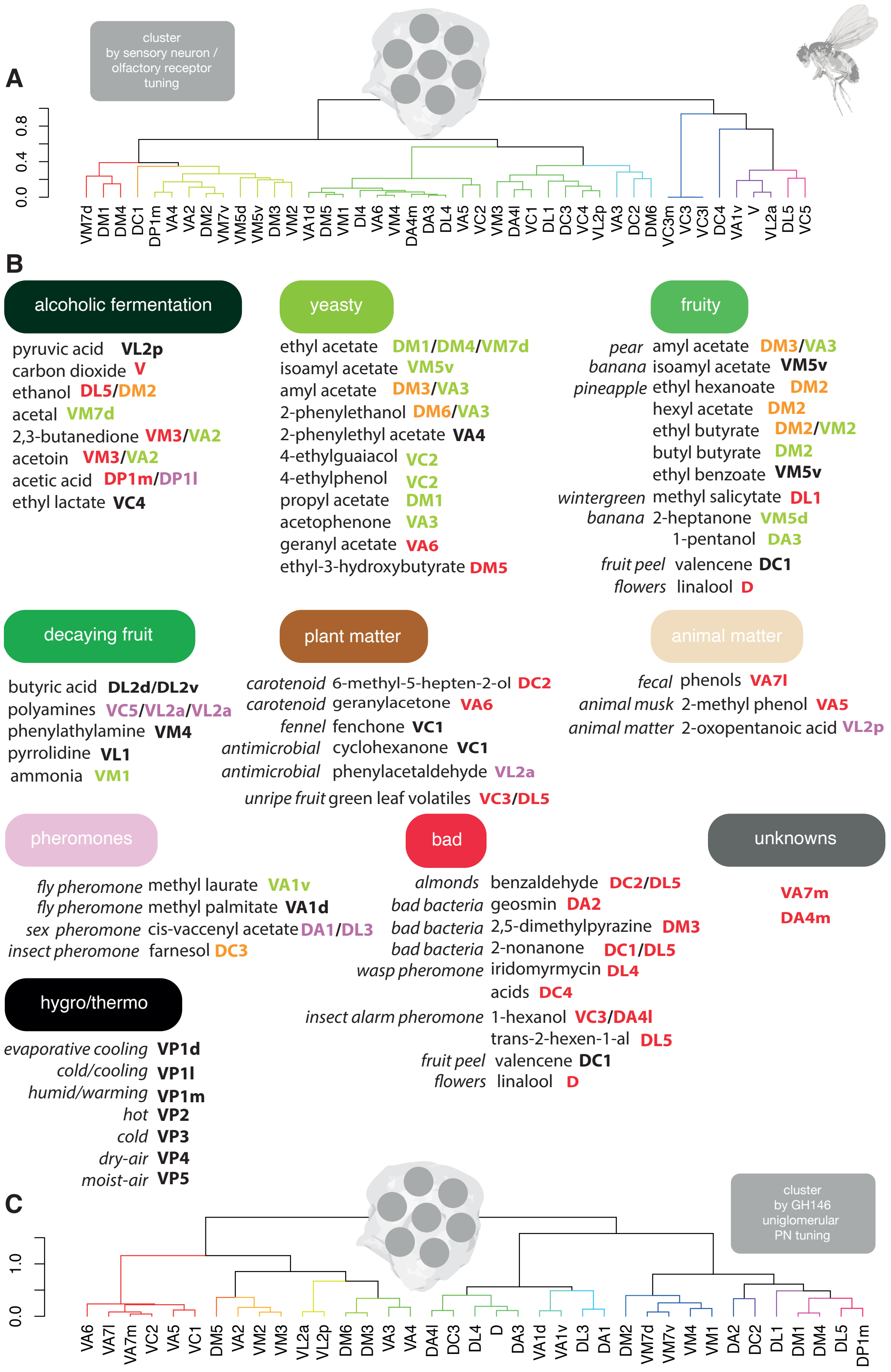


**Figure S7: Glomeruli valences and odours scenes, Related to Figures 2 and 6. A** Hierarchical clustering (Ward's method, arbitrary height cut off for colouring at 0.3) of manipulated odour response data for ORs and ORNs from the cross-study normalised DoOR 2.0 database. We used the OR to uPN transform described in Olsen & Wilson [S10] to estimate uPN responses and then calculated their Gower distances, a method chosen to deal with large amounts of missing data. **B** Segregating odour spaces based on a literature review: most prominently using PubChem, the DoOR database, literature summaries from Huoviala *et al.* [S11] and Mansourian & Stensmyr [S12] as well as other recent work [S8, S12–S16]. Example strong ligands for glomeruli are given and in places their occurrence in nature (where this is clear and non-repetitive). Glomeruli are coloured by putative valences (appetitive green , aversive red , black unknown, based on estimates from the literature [S15], purple complex/dimorphic, orange disputed). **C** Hierarchical clustering (Ward's method, arbitrary height cut off for colouring at 0.3) of odour response data from Ca2+ responses in the dendrites of *Drosophila*, uniglomerular GH146 positive PNs [S17], after min-max normalisation and calculating their cosine distances.

## Supplemental References


S1. Ito, M., Masuda, N., Shinomiya, K., Endo, K. & Ito, K. Systematic analysis of neural projections reveals clonal composition of the Drosophila brain. *Curr. Biol.* **23,** 644–655 (Apr. 2013).

S2. Wong, D. C., Lovick, J. K., Ngo, K. T., Borisuthirattana, W., Omoto, J. J. & Hartenstein, V. Postembryonic lineages of the Drosophila brain: II. Identification of lineage projection patterns based on MARCM clones. *Dev. Biol.* **384,** 258–289 (Dec. 2013).

S3. Lovick, J. K., Ngo, K. T., Omoto, J. J., Wong, D. C., Nguyen, J. D. & Hartenstein, V. Postembryonic lineages of the Drosophila brain: I. Development of the lineage-associated fiber tracts. *Dev. Biol.* **384,** 228–257 (Dec. 2013).

S4. Yu, H.-H., Awasaki, T., Schroeder, M. D., Long, F., Yang, J. S., He, Y., Ding, P., Kao, J.-C., Wu, G. Y.-Y., Peng, H., *et al.* Clonal Development and Organization of the Adult Drosophila Central Brain. *Curr. Biol.* **23** (Mar. 2013).

S5. Münch, D. & Galizia, C. G. DoOR 2.0–Comprehensive Mapping of Drosophila melanogaster Odorant Responses. *Sci. Rep.* **6,** 21841 (Feb. 2016).

S6. Schneider-Mizell, C. M., Gerhard, S., Longair, M., Kazimiers, T., Li, F., Zwart, M. F., Champion, A., Midgley, F. M., Fetter, R. D., Saalfeld, S., *et al.* Quantitative neuroanatomy for connectomics in Drosophila. *Elife* **5** (Mar. 2016).

S7. Frechter, S., Bates, A. S., Tootoonian, S., Dolan, M.-J., Manton, J., Jamasb, A. R., Kohl, J., Bock, D. & Jefferis, G. Functional and anatomical specificity in a higher olfactory centre. *eLife* **8** (eds VijayRaghavan, K. & Grunwald Kadow, I. C.) e44590. ISSN: 2050-084X (May 2019).

S8. Jeanne, J. M., Fişek, M. & Wilson, R. I. The Organization of Projections from Olfactory Glomeruli onto Higher-Order Neurons. *Neuron* **98,** 1198–1213.e6 (June 2018).

S9. Caron, S. J. C., Ruta, V., Abbott, L. F. & Axel, R. Random convergence of olfactory inputs in the Drosophila mushroom body. *Nature* **497,** 113–117 (May 2013).

S10. Olsen, S. R. & Wilson, R. I. Lateral presynaptic inhibition mediates gain control in an olfactory circuit. *Nature* **452,** 956–960 (Apr. 2008).

S11. Huoviala, P., Dolan, M.-J., Love, F. M., Frechter, S., Roberts, R. J., Mitrevica, Z., Schlegel, P., Bates, A. S., Aso, Y., Rodrigues, T., *et al.* Neural circuit basis of aversive odour processing in Drosophila from sensory input to descending output. *bioRxiv* (2018).

S12. Mansourian, S. & Stensmyr, M. C. The chemical ecology of the fly. *Curr. Opin. Neurobiol.* **34,** 95–102 (Oct. 2015).

S13. Dweck, H. K. M., Ebrahim, S. A. M., Thoma, M., Mohamed, A. A. M., Keesey, I. W., Trona, F., Lavista-Llanos, S., Svatoš, A., Sachse, S., Knaden, M., *et al.* Pheromones mediating copulation and attraction in Drosophila. *Proc. Natl. Acad. Sci. U. S. A.* **112,** E2829–35 (2015).

S14. Mohamed, A. A. M., Retzke, T., Das Chakraborty, S., Fabian, B., Hansson, B. S., Knaden, M. & Sachse, S. Odor mixtures of opposing valence unveil inter-glomerular crosstalk in the Drosophila antennal lobe. *Nat. Commun.* **10,** 1201 (Mar. 2019).

S15. Badel, L., Ohta, K., Tsuchimoto, Y. & Kazama, H. Decoding of Context-Dependent Olfactory Behavior in Drosophila. *Neuron* **91,** 155–167 (July 2016).

S16. Galizia, C. G., Münch, D., Strauch, M., Nissler, A. & Ma, S. Integrating heterogeneous odor response data into a common response model: A DoOR to the complete olfactome. *Chem. Senses* **35,** 551–563 (Sept. 2010).

S17. Badel, L., Ohta, K., Tsuchimoto, Y. & Kazama, H. Decoding of Context-Dependent Olfactory Behavior in Drosophila. *Neuron* **91,** 155–167 (2016).
